# Inhibitor Design Strategy for Myostatin: Dynamics and Interaction Networks Define the Affinity and Release Mechanisms of the Inhibited Complexes

**DOI:** 10.3390/molecules28155655

**Published:** 2023-07-26

**Authors:** Dóra Nagy-Fazekas, Zsolt Fazekas, Nóra Taricska, Pál Stráner, Dóra Karancsiné Menyhárd, András Perczel

**Affiliations:** 1 Laboratory of Structural Chemistry and Biology, Institute of Chemistry, Eötvös Loránd University, Pázmány Péter sétány 1/A, H-1117 Budapest, Hungary; fdora@student.elte.hu (D.N.-F.);; 2 Hevesy György PhD School of Chemistry, Institute of Chemistry, Eötvös Loránd University, Pázmány Péter sétány 1/A, H-1117 Budapest, Hungary; 3 ELKH-ELTE Protein Modeling Research Group, Eötvös Loránd Research Network (ELKH), Institute of Chemistry, Eötvös Loránd University, Pázmány Péter sétány 1/A, H-1117 Budapest, Hungary

**Keywords:** negative regulator of muscle mass, myostatin inhibition, growth differentiation factor 11 (GDF11), Gaussian–Mahalanobis mean

## Abstract

Myostatin, an important negative regulator of muscle mass, is a therapeutic target for muscle atrophic disorders such as muscular dystrophy. Thus, the inhibition of myostatin presents a strategy to treat these disorders. It has long been established that the myostatin prodomain is a strong inhibitor of the mature myostatin, and the minimum peptide of the prodomain—corresponding to the α_1_-helix of its lasso-region—responsible for the inhibitory efficiency was defined and characterized as well. Here we show that the minimum peptide segment based on the growth differentiation factor 11 (GDF11), which we found to be more helical in its stand-alone solvated stfate than the similar segment of myostatin, is a promising new base scaffold for inhibitor design. The proposed inhibitory peptides in their solvated state and in complex with the mature myostatin were analyzed by in silico molecule modeling supplemented with the electronic circular dichroism spectroscopy measurements. We defined the Gaussian–Mahalanobis mean score to measure the fraction of dihedral angle-pairs close to the desired helical region of the Ramachandran-plot, carried out RING analysis of the peptide-protein interaction networks and characterized the internal motions of the complexes using our rigid-body segmentation protocol. We identified a variant—11m2—that is sufficiently ordered both in solvent and within the inhibitory complex, forms a high number of contacts with the binding-pocket and induces such changes in its internal dynamics that lead to a rigidified, permanently locked conformation that traps this peptide in the binding site. We also showed that the naturally evolved α_1_-helix has been optimized to simultaneously fulfill two very different roles: to function as a strong binder as well as a good leaving group. It forms an outstanding number of non-covalent interactions with the mature core of myostatin and maintains the most ordered conformation within the complex, while it induces independent movement of the gate-keeper β-hairpin segment assisting the dissociation and also results in the least-ordered solvated form which provides extra stability for the dissociated state and discourages rebinding.

## 1. Introduction

Myostatin, or growth differentiation factor 8 (GDF8), is a transforming growth factor β (TGF-β) family member that is an important regulator of skeletal muscle growth [1]. The TGF-β superfamily includes a large number of signaling proteins which play important roles in regulating embryonic development and maintaining tissue homeostasis [2]. Myostatin acts as a negative regulator of muscle growth [1,3]; thus, the systemic overexpression of myostatin causes muscle loss [4,5], while the deletion or mutation of the myostatin gene causes the increase of skeletal muscle mass [1,5]. The inhibition of myostatin is currently sought for the treatment of skeletal muscle-related disorders such as muscular dystrophy, sarcopenia and cancer cachexia [6,7]. In addition, recently, it was shown that myostatin is involved in the regulation of the physiology and pathology of ovarian reproductive functions as well and blocking the myostatin signaling pathway can be a potential therapy for ovulation disorders also [8,9].

**Figure 1:**
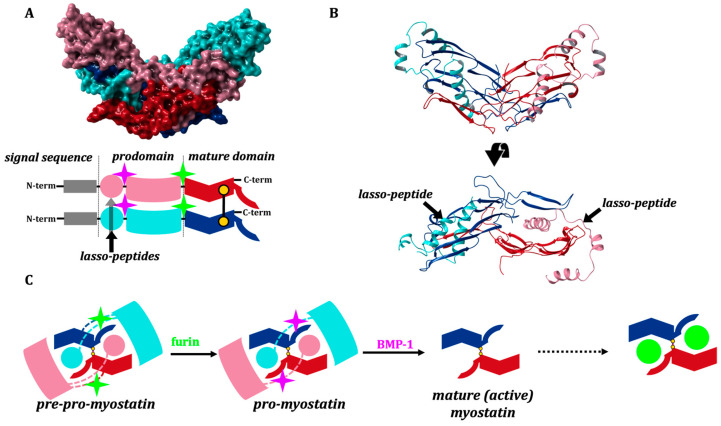
(**A**) 3D and domain structure of myostatin (GDF8), indicating the cleavage sites of furin-like protease (green) and BMP-1 (lila) and the position of the disulfide bond. (**B**) 3D model was created based on crystal structure 5NTU [13] and the GDF8 model of AlphaFold2 [14] and colored according to the schematic view of the domain structure. The structure of mature myostatin blocked by the lasso-peptide. (**C**) Activation path of myostatin: cleavage by a furin-like protease leads to the pro-myostatin form, while cleavage by BMP-1 affords the mature active state.

Myostatin, similar to many TGF-β family members, emerges in a homodimeric precursor form, linked by a disulfide bond, and goes through two sequential proteolytic steps to reach the fully activated form. First it is cleaved by a furin-like protease [3,10], after which myostatin circulates in the blood in a latent form as a non-covalently bound propeptide (pro-myostatin). A second cleavage step by the bone morphogenetic protein-1 (BMP-1) yields the mature, signaling C-terminal domain and prompts the release of the non-catalytic N-terminal prodomain [3,10,11,12]. The N-terminal prodomain thus functions as the natural inhibitor of the mature fragment, shielding its potent, receptor-binding inner surfaces by latching a helix-loop-helix-loop-helix motif (the so-called “lasso-peptide”) into and over the receptor-binding groove (Figure 1).

Mature myostatin is inhibited by different glycoproteins such as follistatin-like 3 and the TGF-β1 proteins, binding to the receptor binding site by their N-terminal α-helical region [4,7,15]. Active myostatin is inhibited by other proteins as well such as follistatin [16] and WFIKKN1-2 [17]. However, these proteins inhibit other growth factors as well, such as growth differentiation factor 11 (GDF11) [3,16,17,18].

The most specific myostatin inhibitor known today is the myostatin-prodomain, but even this interaction is not very strong [10,11,12]. This is not surprising, since the myostatin lasso-segment was designed by nature to rapidly dissociate from the mature core of myostatin after it is cleaved from the prodomain, allowing activation of the growth factor and its subsequent association with its target receptors (Figure 1C).

It has become a common strategy for inhibitor design to mimic binding of the physiologically recognized partners—thus using short helical segments carrying sequences similar to that of the lasso-helix—to block myostatin action. However, when designing potent myostatin inhibitors, these specific sequences need to be optimized since nature intended them as quick departers not as strong binders, or a better candidate scaffold needs to be found.

Myostatin and GDF11 are in a close relation within the TGF-β family [7,18], since their mature forms are over 94% identical, while their N-terminal prodomains show a more modest ~52% identity [19,20]. Despite their high sequential similarity, structural comparison has shown that these proteins have several differences, mostly localized at the interfaces and loop regions, which are critical for the interactions with receptors. The biological role of GDF11 is less understood than the role of myostatin; however, it is known that GDF11 is a more potent activator of SMAD2/3 and signals more effectively through the type I activin-like receptor kinase receptors ALK4/5/7 than myostatin [20]. According to recent results, GDF11 also has a role in the regulation of the central nervous system formation and in maintaining its integrity during the lifetime [21].

It has been shown that the segment corresponding to the N-terminal first helix of the lasso-peptide of the myostatin prodomain can be used for the selective inhibition of the mature myostatin [4,22]. Hayashi et al. studied inhibition of human myostatin based on such a short peptide sequence derived from the mouse myostatin prodomain [23]. They identified a 24 amino acid-long minimum sequence that is important for selective inhibition of the mature myostatin and synthesized a series of mutant peptides and demonstrated their antimyostatin activities [4,22,23,24,25,26,27]. In recent years, several protein inhibitors have been reported [28,29], and some of these inhibitors underwent initial clinical trials [29,30] but did not progress further.

In the present study, based on molecular dynamics simulations and electronic circular dichroism (ECD) spectroscopy measurements, we show that the minimum sequence of the GDF11 prodomain is a better inhibitor scaffold against myostatin. Our results revealed that the solution-state form of even those peptides that assume a helical conformation within the complex prior to cleavage loses most of their helicity when liberated from the mature core. Therefore, we set out to increase their inhibitory potential by increasing their stand-alone helicity and optimizing their sequence in a manner that would improve local contacts within the complex. We show that the minor changes on the minimal peptide sequence have high impact on the stability of the whole complex. In addition, the stability of the complex also depends on the nature and strength of the interaction between the peptide and a beta-hairpin of mature myostatin, which may function as a controlled gate of the complex. We provide a protocol that can be applied for further GDF8/DGF11 inhibitory peptide design and development.

## 2. Results and Discussion

In the present work, we aimed to examine the interaction between the mature myostatin domain and the prodomain lasso-helices and the structure and dynamics of the fully cleaved and inhibited mature complex (see Figure 1C).

### 2.1. Solution Structure of the Lasso-Peptides of Myostatin (GDF8), GDF11 and Their Variants

**Figure 2:**
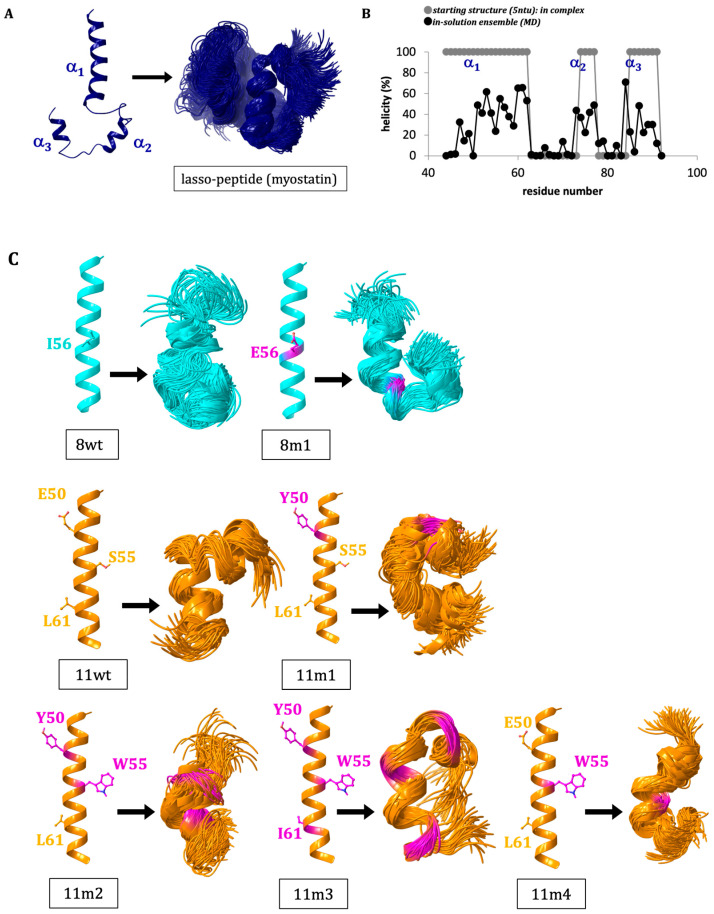
(**A**) The starting structure (extracted from the crystal structure of the pro-myostatin complex (5NTU)) and the derived conformational ensemble for the 51-residue lasso-peptide of myostatin (showing the mid-structures of the clusters that together represent over 90% of the snapshots—thus the number of structures shown corresponds to the conformational heterogeneity of each system). (**B**) Change in the helicity of the 44–94 segment upon removal from the pro-myostatin complex. (**C**) The starting structure and derived ensembles of the simulations carried out for N-terminal 1st helix of the lasso-peptide (α_1_) of the wild-type growth factors myostatin/GDF8 (8wt) and GDF11 (11wt) and designed mutant peptides (8m1, 11m1, 11m2, 11m3 and 11m4).

First, we aimed to investigate our assumption that the lasso-peptide regions of the growth factors lose most of their highly ordered helical structure after the release from the mature core. Molecular dynamic (MD) simulations were carried out to analyze the conformational ensembles of different segments of the lasso-helix by themselves (without the myostatin prodomain or the mature myostatin-core). In myostatin, the lasso-peptide spans a 51-residue segment that is broken into 3 helices and connecting loops. The longest N-terminal helix is formed by residues 41–64. We found that in solution, the helical region of this segment is reduced to the 52–64 section, while the second two helices also considerably deteriorate. Using the default measure of the GROMACS simulation package, the average helical content is reduced from 61.2% (along the 44–94 residue range of the lasso-peptide (the region forming direct contacts with the core)) to 23.1% (Figure 2A,B). When the N-terminal helix (residues 44–66) was simulated in itself, even lower, 15.5% helicity was found. We also carried out a simulation using the sequence of the GDF11 prodomain (11wt), and we found a considerably higher helicity for this system: the average helicity for the 44–66 segment was found to be 29.6%. Based on this finding, the α_1_-helix of the lasso-peptide of GDF11 (11wt) proved to be a better candidate for inhibitor optimization (Figure 2C).

**Figure 3:**
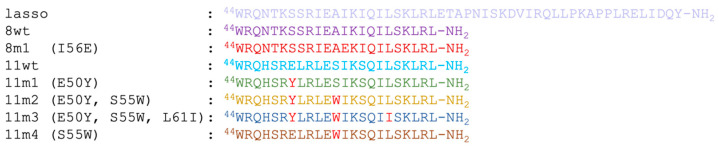
Amino acid sequences of the designed peptides. Light-red-colored amino acids show the designed mutations from peptide to peptide.

To better understand the factors influencing the inhibitory potential of various α_1_-mimics over the active myostatin domain, we designed different mutant peptides using the wild-type variants (8wt and 11wt) as scaffolds (Figure 2). A single mutant was constructed from 8wt, that of 8m1, carrying the I56E mutation. This mutation was shown to significantly increase the activity of myostatin [3], leading to dissociation of the pro-myostatin complex even in absence of the second cleavage step. Glu in position 56 was proposed to disrupt a hydrophobic interaction center between the α_1_-helix and the prodomain [3], so in this work the 8m1 peptide was used as a negative control. In the first GDF11 peptide mutant (11m1), a glutamic acid at position 50 was changed to tyrosine, because this Tyr has been indicated earlier as playing an important role in the inhibition of the active GDF8 through increasing the helicity of the peptide [4]. In the case of 11m2, an additional S55W switch was introduced, which is also known to help in achieving better inhibition [26]. 11m3 contains a third I61L mutation. Ile and Leu are both capable of significantly contributing to the stability of a hydrophobic core [4,26], but with slightly different topology which might fine-tune the orientation and fit of the tightly packed interaction surfaces. Finally, the 11m4 variant contains the S55W switch in itself. The different peptides’ amino acid sequences can be seen in Figure 3.

**Figure 4:**
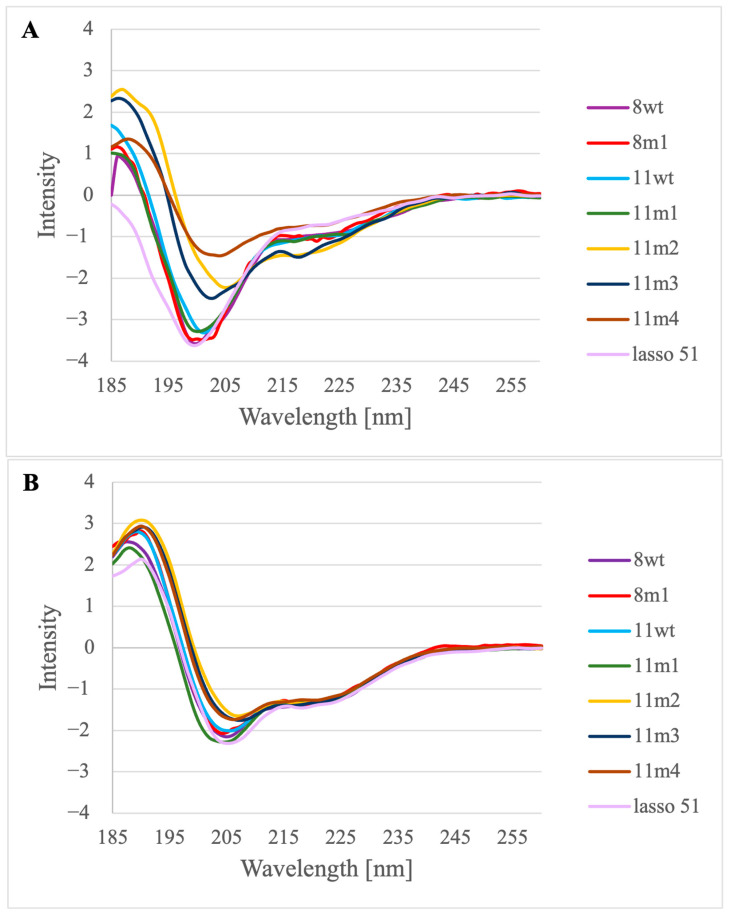
FUV-ECD measurements of the peptides 8wt, 8m1, 11wt, 11m1, 11m2, 11m3, 11m4 and lasso 51: (**A**) without added TFE at 25 °C and (**B**) with 10% TFE added at 25 °C.

The peptides were synthetized applying a continuous flow solid phase peptide synthesis technique described recently by Farkas et al. [31,32]. Purification was carried out by Reversed Phase-High Performance Liquid Chromatography (RP-HPLC), and the peptide containing fractions were lyophilized. Mass spectrometry (MS) analysis was also performed in the case of each peptide to demonstrate the purity of the samples (Supplementary Information Figures S1–S8). We examined the structure of the purified samples by far-UV ECD (FUV-ECD) measurements (preparing samples that contained the peptides in the 30–60 µM concentration range) (Figure 4A,B) [33,34]. Although the baseline was extracted from each measured protein spectrum and the raw ellipticity unit was recalculated, the intensity in the case of the 11m2 peptide is still outstanding, possibly due to micro-aggregation, so in order to focus on the shape of the spectrum, the measured values were uniformly scaled using the methods described in [35,36].

First, we established that the 51-residue long lasso-peptide of myostatin has a low content of ordered structure when it is not in the complex with the myostatin. Comparing the spectra of the minimum length—or α_1_—peptides, we conclude that the wild-type sequences (8wt and 11wt) and the negative control (8m1) show—as expected—very low content of ordered structure. In these spectra, a negative band characteristic of disordered proteins can be seen at around 200 nm, which has a weaker negative shoulder at 220 nm. The peptide 11m1 shows some structural ordering, since this characteristic band is less negative, while the peptides 11m2, 11m3 and 11m4 have the most helical form—in line with our intent. In these spectra, as it is typical for helical peptides, a positive band appears at around 192 nm, while a negative band appears at around 208 nm, and perhaps a weak negative band is also visible around 220 nm as well (Figure 4A) [35,36].

To quantify the secondary structural content of the solution state of the peptides, deconvolution of the spectra was carried out using the CCA+ protocol [37]. The derived helical content values (Table S1) confirm that while 11wt is indeed more helical than 8wt (23.3% and 14.6%, respectively), both present low helicity values which are considerably increased in the 11m2, 11m3 and 11m4 variants (70.2%, 54.0% and 58.5%, respectively). Surprisingly, the 11m1 peptide, which carries the E50Y mutation which has effectively boosted the helicity of the minimal peptide derived from mouse GDF8, is among the least helical (15.8%) of those studied by us, according to ECD.

The peptides dissolved in water were measured directly and with 10% trifluoroethanol (TFE) added to the samples, to test whether they show tendency to form helices (Figure 4B). It is visible from the spectra of the TFE containing samples that the C type spectrum typical to the helical peptides appears in all cases. All the spectra were obtained at three different temperatures (5–25 °C) to see whether temperature influences the peptide structure. However, it seems that these peptides are not sensitive to moderate temperature changes (Figures S9 and S10).

Since the 11wt peptide is a partially disordered peptide according to the measurements, we further examined whether this peptide could lose its structure completely when it is subjected to higher temperatures. Interestingly, this is not the case, even at 90 °C, as an ordered core remains (Figure S11), confirming that this sequence is a promising basis for inhibitor design.

While the measured (ECD and CCA+) and calculated (MD simulation) helicity values were in agreement concerning the low helicity of the wild-type sequences and the approximate effect of the mutations, definite discrepancies could also be seen between the two. Since the lyophilized and re-solvated peptides used in our measurements could not completely be separated from TFA and additional ions were added to the solution during the setting of neutral pH of the samples, the conditions of the simulations were accordingly modified from 0.15 M NaCl to a 0.5 M NaCl medium. These new simulations reproduced the measured helical tendencies more satisfactorily (Table S2, Figures S12 and S13) and thus verified our calculation protocol, but a comparison of the free and complexed states of the peptides and their detailed analysis was carried out using the simulated ensembles containing physiological salt concentration (0.15 M).

**Figure 5:**
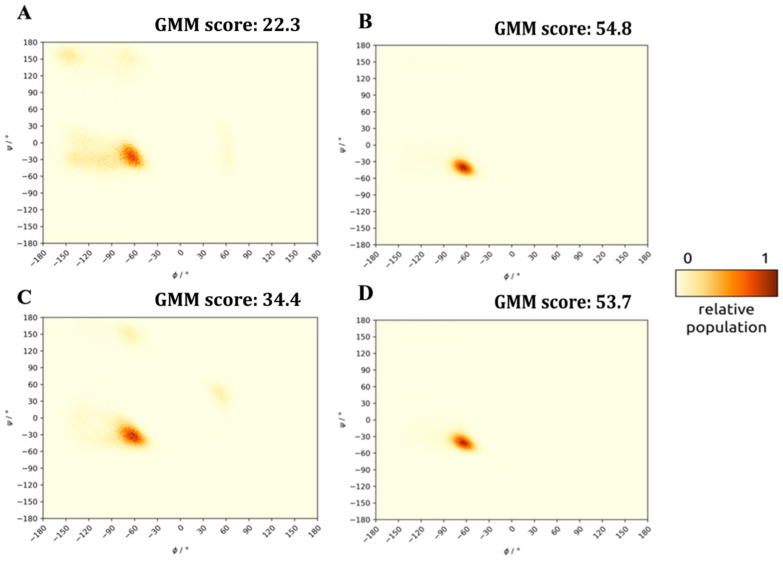
Ramachandran plots and GMM scores of snapshots of the last 500 ns MD simulation of peptides solvated by themselves and within the cleaved, but un-dissociated (latent) complex (c_NaCl_ = 0.15 M). (**A**) stand-alone 8wt. (**B**) 8wt in complex. (**C**) stand-alone 11wt. (**D**) 11wt in complex.

The disagreement between the calculated and measured helicity values also prompted us to introduce further metrics for describing helical content of the structural ensembles created by the snapshots of the equilibrated MD trajectories. First, we created Ramachandran plots based on the backbone Φ_i_, Ψ_i_ angle-pairs of all snapshots of the final 500 ns of the MD simulation of each peptide. This view differs from the helicity measure calculated previously by relying solely on the backbone conformation, while the helicity measure also considers whether the characteristic H-bond pattern of α-helices (backbone NH_i_…C=O_i−4_) is able to form. Thus, the topology of the Ramachandran plots indicates a propensity for helical (or any other) secondary conformation instead of measuring if signature H-bond motifs are actually present or not. In Figure 5A,C (and Figures S12 and S13), it can be seen that all free (uncomplexed) peptides show a high right-handed α-helical propensity shown by the significant population of structures clustered near Φ_i_ = −65°, Ψ_i_ = −30°, the center of the classical α-helical distribution. However, distributions centered on the α-helical conformation are spread widely, and backbone Φ_i_, Ψ_i_ pairs sampling turns or even β-stranded secondary structures were also detected. The extent of the deviation from the α-helical backbone structures indicates the energy needed to induce the helical fold. To measure the distance of each Φ_i_, Ψ_i_ pair from the distribution of classified α-helices found in crystal structures, first we established the center and spread of helical backbone values of high-resolution, non-homologous protein structures of the Protein Data Bank (PDB) and then calculated the Mahalanobis distance of each Φ_i_, Ψ_i_ pair found in our MD simulations from this distribution (affording a Gaussian–Mahalanobis mean (GMM) score: the fraction of dihedral angle-pairs close to the desired helical region) (See Material and Methods (Section 3) for further details). Evaluation of both the helicity values and the GMM scores of MD ensembles of the stand-alone peptides solvated in 0.15 M physiological NaCl solution resulted in a helicity ordering of 11m1 > 11wt >> 11m2~8m1 > 11m4 > 11m3 >> 8wt. Thus, the natural, wild-type α_1_ lasso-peptide (8wt) proved to be the least helical of all studied variants, while 11wt and 11m1 were found to be highly helical. This finding also reaffirms our previous conclusion that the 11wt sequence is a good scaffold for myostatin inhibitor design and that the E50Y mutation suggested by Hayashi et al. [4] increases the helicity of the human GDF11-derived peptide significantly, as it was also seen in case of the minimal sequence peptide designed based on mouse myostatin.

The low helicity of the wild-type sequence (8wt) is in line with its physiological role which requires the fast release and diminished rebinding capacity of the lasso-segments. Relaxing from the helical conformation once removed from the complex contributes to function: after release, the re-association of the 8wt peptide segment with the mature core of myostatin can only proceed via an induced helicalization step. Possessing a fluctuating, flexible solvated structure is also favorable energetically, providing entropic stabilization for the dissociated state—achieving which is the ultimate goal of the myostatin activation process. However, for an inhibitor, the reverse would be required; thus, a higher helical content in the stand-alone form would be advantageous.

### 2.2. Structure of the Inhibited Complex: Helicity

After studying the solvated state of the α_1_-helix variants, we carried out an MD simulation of the dimeric non-covalent complex of myostatin with each peptide, the inhibited form of the mature core of myostatin (see Figure 1C). The calculated helicity of the peptides in the peptide–myostatin complexes (based on the MD simulations) shows considerably increased GMM scores as compared with that of the stand-alone peptides (see Figure 5B,D and Figure S14). In the complex form, the peptides readily form helices, an effect probably driven by the structure-stabilizing nature of the active myostatin domain. While all myostatin-bound peptides are helical within the complex, the 11m3, 11m4 and 8wt sequences are the most helical of all. It is interesting to note that the original, physiological α_1_-helix of the prodomain (8wt) is not only the one that is most relaxed in the solvated form but also one of the most ordered within the protein interior of myostatin core-complex. Nature seems to have optimized this segment for maximal stability within the complexes of the non-processed pre-/pro-states of the activation cascade but also for maximal instability in its detached form, to guarantee both its secure binding in the self-inhibited state and its fast and complete release when the growth signal arrives. It is also important to note, that variants of the 11wt sequence can achieve as high or even higher helicity within the complex, resulting in highly stable inhibited states.

### 2.3. Structure of the Inhibited Complex: Interaction Networks

**Figure 6:**
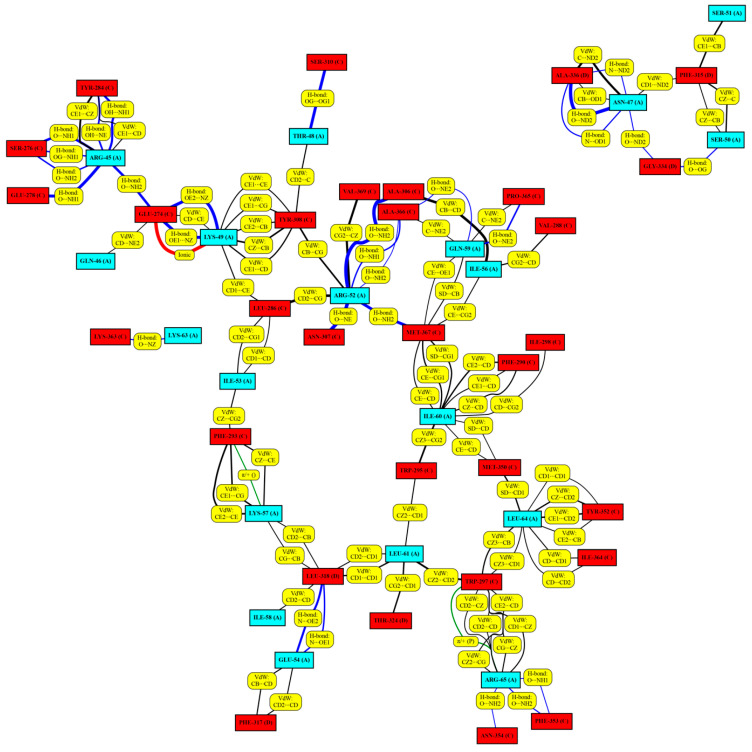
The RING analysis map of the simulated peptide 8wt in complex (the RING maps of the remaining peptides can be found in the Supplementary Information file Figures S15–S20). The amino acids in cyan boxes represent the α-helix peptide chain members, while the amino acids in red boxes represent the mature myostatin chain members interacting with the α-helix peptide chain members. Yellow boxes explain the different interaction types between the individual amino acid pairs. Lines of different thickness and color indicate the different interaction types between the individual amino acid pairs: **black**: Van der Waals interaction; **blue**: H-bond; **green**: Cation-π and π-π stack and **red**: ionic interaction. The thickness of the lines indicates the strength of the interaction; the thicker the line, the greater the percentage of the frames where the interaction is present.

RING interaction graphs [38], showing all non-covalent interactions between the α_1_-peptides and mature myostatin (Figure 6 and Figures S15–S20), were constructed to map the entire peptide–host interactome. Numerical data were also extracted from these graphs, such as the average (with respect to all frames) number of interactions of specific interaction types (salt bridges, H-bonds, Van der Waals interactions, etc.), and the average number of interactions belonging to specific residues (Table 1) was calculated.

This way, we were able to identify key residues and interactions forming between the protein partners.

According to the RING analysis, the peptide with the most in-complex hydrogen bonds is 8wt (14.5 per peptide, on average). The smallest mean value of the number of H-bonds was obtained in the case of the 11m4 peptide. Interestingly, as we incorporate the mutations into the 11wt peptide, we do not achieve larger H-bond numbers than in the wild-type peptide. This emphasizes how co-evolution of these segments have perfected their interaction surfaces.

When the helix residues are ordered according to their average contact numbers, several shared interaction hot-spots can be identified. As an example, the K49 residue in 8wt and 8m1 or the R49 residue in the 11wt and its mutants always form a salt-bridge with the E274 of the active myostatin domain. The residue R45 is also in contact with E274, along with E278 and Y284, the latter one of which forms Van der Waals interactions with the aliphatic part of the arginine. This residue only loses its coordinating nature in 11m1, where it orients its side chain towards the bulk solvent. Another important arginine is R52, which holds on to the backbone of A306, N307 and sometimes M367 and also interacts hydrophobically with the aromatic ring of Y308. L64 commonly participates in the formation of a hydrophobic core enclosed by W297, I298, M350, Y352 and, loosely, I364. Lastly, K57 also appears in 8m1, 11m1 and 11m4 as a key inter-chain interacting residue, since, besides forming an intra-chain salt-bridge with E54, it also serves as a hydrophobic anchor for residues F293 and W295 creating crosstalk between the two halves of the dimeric complex.

However, important mutation-induced differences can also be observed in the interaction networks of the different complexes. When compared to the 8wt peptide, the I56E mutation of 8m1 affects several residues, namely causing the disappearance of R52 and N47 from the top five residues with the most contacts and the appearance of R65 and K57 on this list. The newly introduced negative charge on E56 disturbs the electrostatic pattern of the peptide, removes the H-bond network formed by R52 and the aforementioned residues and instead forces R52 to create a salt-bridge with the C-terminal carboxylate of S375. This partially unfolds the helical structure of the peptide between the interval W44–R52, which is not beneficial for the myostatin binding, as reported before [3].

**Figure 7:**
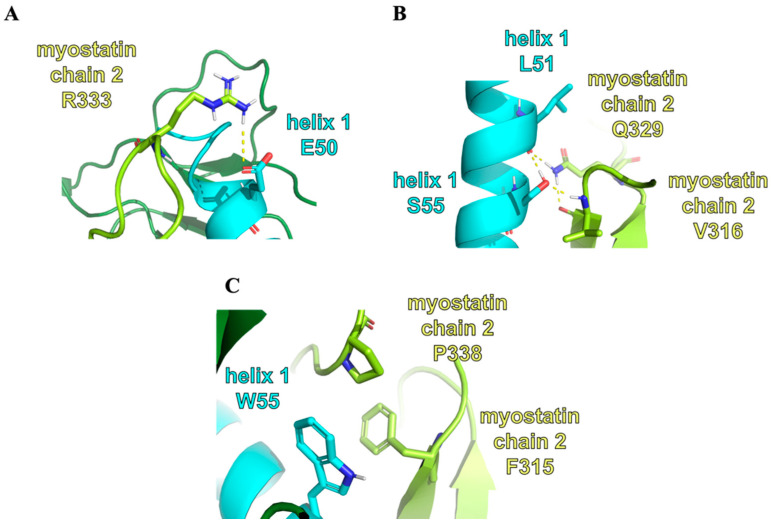
The representation of different key interactions and residues: (**A**) The representation of the interaction between the residue E50 of the 11wt helix 1 and the R333 residue of the myostatin chain 2. (**B**) The representation of the interaction between the residues L51 and S55 of the 11wt helix 1 and the V316 and Q320 residues of the myostatin chain 2. (**C**) The representation of the interaction between the residue W55 of the 11m3 helix 1 and the F315 and P338 residues of the myostatin chain 2.

The differences between the interaction networks of the 8wt and 11wt complexes are interestingly minimal, despite their large sequential difference. Since the myostatin and GDF11 mature core-domains are nearly identical, the α_1_ helices of the respective complexes are nature’s two different solutions to optimizing the interaction with the almost analogous binding surfaces. The difference in sequence indicates that more than one feature might have been simultaneously optimized. In case of the 8wt and 11wt peptide segments, this further attribute might be the solution state the propensity to unfold from the ordered helical conformation—which we have shown to be quite different for the two—which might affect the kinetics and effectivity of dissociation and through this the functioning of the two different signaling systems. However, some characteristic differences do appear in the binding mode of these peptides too. The importance of L51 is unique to the 11wt peptide (Figure 7), for example. Here, the backbone carbonyl of this residue is stably held by the side chain amide of Q329, while the side chain of the leucine forms a hydrophobic contact with F315. In the other GDF11 mutant peptide complexes, as well as the GDF8 complexes, only the F315 interaction remains, while the Q329 residue faces the solvent. The beta-hairpin structured region, where this Q329 residue resides, is of high importance, as our analysis later indicated.

The E50Y mutation is present in the peptides 11m1, 11m2 and 11m3. The wild-type residue, E50, is in a salt-bridge with R333 in 11wt and 11m4, which causes the deformation of the beta-hairpin region V316–H328 (Figure 7). Similarly, the S55 residue in 11wt and 11m1 forms a strong H-bond with the backbone of V316, which is also part of the beta-hairpin structure, while its conformation is also highly limited by the intra-chain H-bond with the L51 backbone. This means that the introduction of the S55W mutation disrupts this H-bond network and puts less strain on the conformation of the hairpin. The mutant tryptophan also fits into the pocket between the hairpin and the opposite side of the myostatin (i.e., the other myostatin chain), resulting in better shape-compatibility. The last mutation, L61I, is close to the C-terminal of the helix. The repositioning of one of the Cδ carbon atoms to the Cγ position mostly affects the behavior of C-terminal L66 residue. It can be observed that while in the 11m2 mutant the L66 residue detaches itself from the rest of the helix, in the 11m3 peptide this C-terminal is more ordered and participates in helix formation. We also wanted to examine the effect of the S55W mutation alone, without the E50Y and L61I mutations. In the 11m4 peptide, the salt-bridge forming effect of E50 can be seen without the hairpin-anchoring effect of S55. This tryptophan only has interacting partners in the 11m3 and 11m4 complexes, namely F315 and P338. This aromatic-proline-aromatic triad is a commonly appearing and stable structural motif in other proteins [39].

### 2.4. Structure of the Inhibited Complex: Dynamics

One of the most characteristic features of the dynamic trajectories of myostatin complexes is that while the association between the mature domain and the complexed helices is tight, the two lobes of the system seem rather free to move with respect to each other. Not only do the two chains maintain a “flutter-of-wings” type fluctuation during the simulations, but characteristic differences can also be seen between the mean and range of the angle of opening formed by the two lobes in the different complexes. This is quite remarkable especially in light of the fact that—in most cases—these systems differ only by a single amino acid of the coordinated ligand. Similarly, when comparing various crystal structures of myostatin in different states and composition, the variability of the opening-angle of the lobes is also apparent.

**Figure 8:**
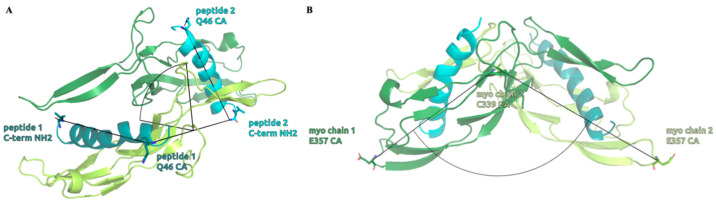
Structural representation of the measured inter-domain dihedral angle (**A**) and opening angle (**B**) numerical descriptors. The inter-domain dihedral angle measures a twisting angle between the two domains through the relative orientation of the peptide helices while the opening angle represents the angle between the two elongated myostatin chains forming the V-shaped complex.

**Figure 9:**
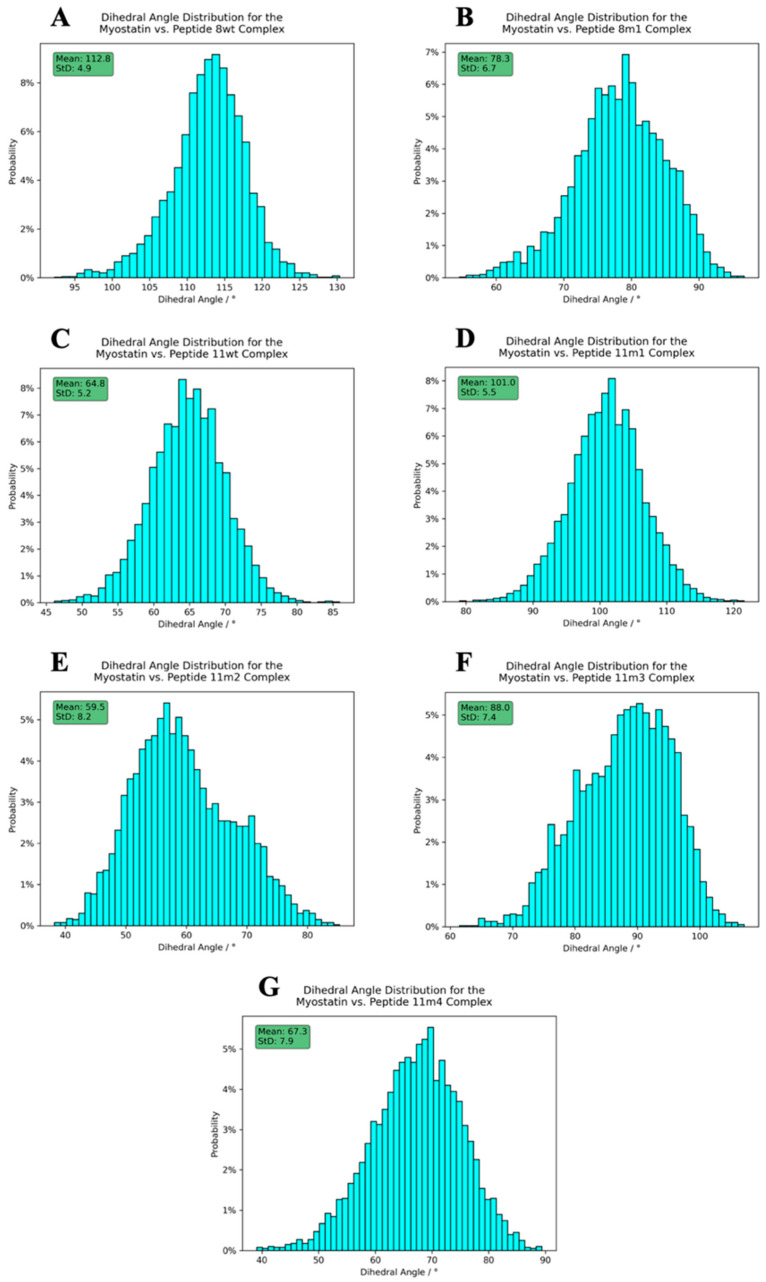
Dihedral angle distribution histograms calculated between the peptides and the active myostatin domain. Panels (**A**–**G**) correspond to peptide 8wt, 8m1, 11wt, 11m1, 11m2 and 11m3, 11m4 complexes, respectively. The average angles, along with their standard deviation, are shown in the upper-left green boxes.

**Figure 10:**
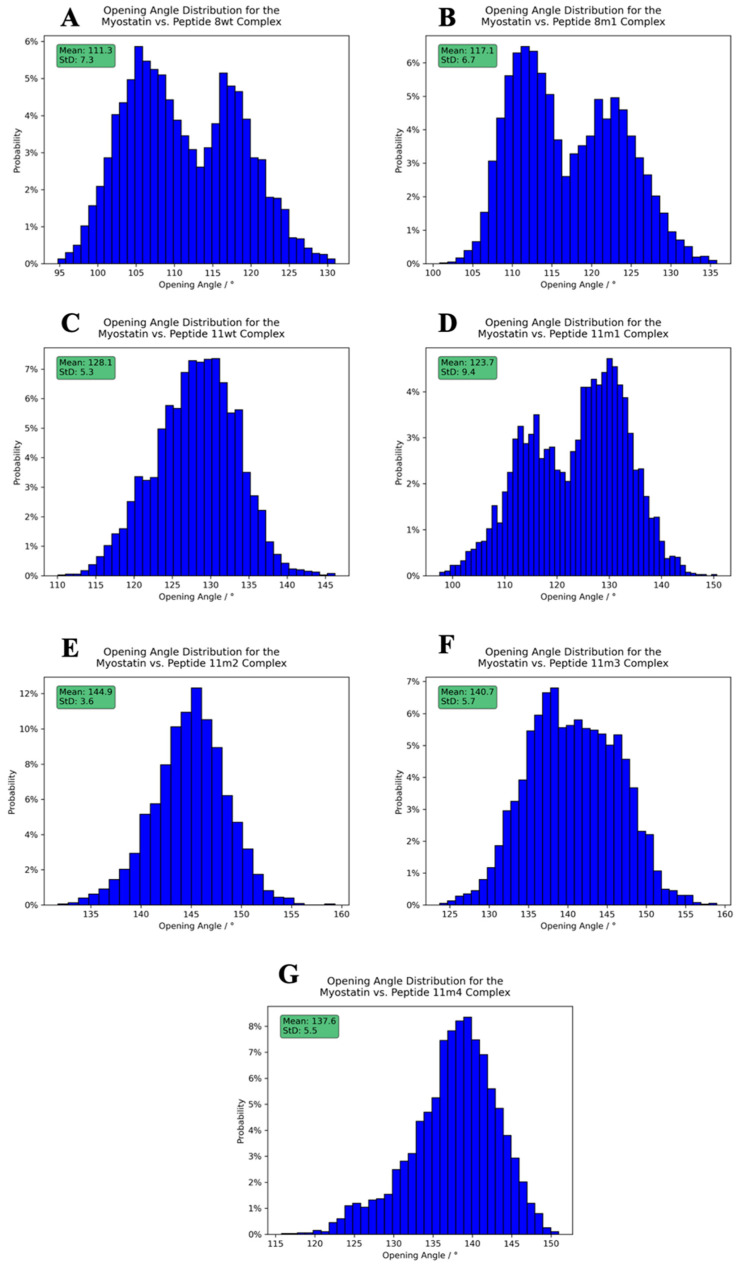
Opening angle distribution histograms calculated between the peptides and the active myostatin domain. Panels (**A**–**G**) correspond to peptide 8wt, 8m1, 11wt, 11m1, 11m2 and 11m3, 11m4 complexes, respectively. The average angles, along with their standard deviation, are shown in the upper-left green boxes.

To investigate the effects of the wild-type and mutant peptides on the dynamic behavior of the complex, we measured the distributions of two structural descriptors, namely the angle enclosed by the myostatin chain 1 E357 CA, myostatin chain 2 C339 CA and myostatin chain 2 E357 CA atoms, which we called the “opening angle” of the complex, and the dihedral angle defined by the peptide 1 C-terminal NH2 N, peptide 1 Q46 CA, peptide 2 C-terminal NH2 N and peptide 2 Q46 CA atoms, to which we refer as the “inter-domain dihedral angle”. These angles are depicted on Figure 8, while the distributions can be seen on Figure 9 and Figure 10, along with their mean and standard deviation values.

The figures clearly show that both the opening angle and the dihedral span a very wide angle range (95.0–160.0° and 40.0–120.0°, respectively), and it is also evident that these values greatly differ in the studied systems. This is a striking observation, since it confirms that even such modest changes as a single Leu→Ile switch cause fundamental changes in the dynamics and the overall shape of the dimeric complexes. The largest mean inter-domain dihedral angle, as well as the smallest mean opening angle, belongs to the peptide 8wt with values 112.8° and 111.3°, respectively. This again emphasizes that the naturally evolved α_1_ sequence is a carefully crafted design that is easily disrupted by mutations. In the experimentally determined myostatin structures, the opening angle varies between 78.0° and 159.5° [20,40,41,42,43], while it ranges from 95.0 to 160.0° in our simulations, indicating that the simulations and the crystal structures sample the same conformational space. Based on the distribution of these angles, we noticed that the mutant 11m2 has extreme values—with respect to the other mutant complexes—both regarding the mean angle values (59.5° for the inter-domain dihedral angle, which is minimal, and 144.9° for the opening angle, which is maximal) and the standard deviation (StD) of the angle values (8.2° for the inter-domain dihedral angle, which is maximal, and 3.6° for the opening angle, which is minimal). These observations prove that the mutations have a large effect on the dynamic behavior of the whole complex and that further analysis is needed to disseminate the exact distorting mechanism of these mutations.

**Figure 11:**
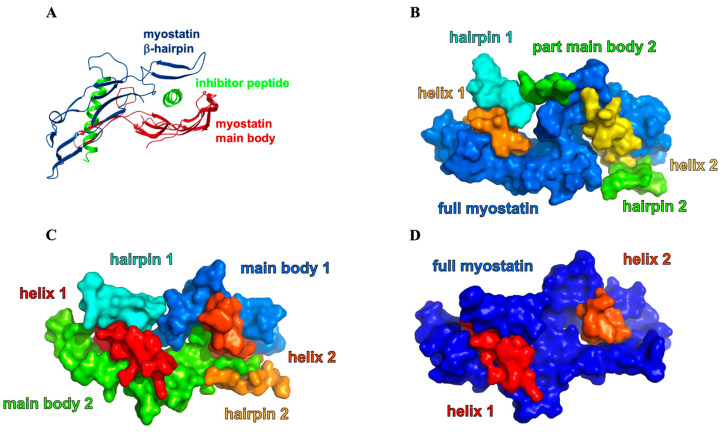
Rigid body segmentation analysis of inhibited myostatin complexes. (**A**) Significant rigid-body segments of the myostatin-inhibitor complexes (colored according to Figure 1C). (**B**) the segmentation of the 8wt-complex shows that β-hairpin gates can move independently from the main-body of myostatin and the ligand helix itself. (**C**) 11m3 peptide–myostatin complex, where the two helices, the two hairpins and the two myostatin main bodies are in all in different segments. (**D**) 11m2 peptide–myostatin complex which shows unique dynamics, since only three segments were detected, namely the two helices and that of the full myostatin mature dimer.

We also carried out Rigid-Body-Segmentation (RBS) [44] analysis (Figure 11 and Figure S21) of the internal movements of the complexes, as reflected in the MD trajectories. We recently described this measure as an approach to identify the different parts of the protein that move in a synchronized manner. RBS decomposes the structure into quasi-rigid segments that fluctuate with respect to each other and in a way that reproduces the fluctuations seen in the trajectory. It does so by clustering the selected atoms (in our case Cα atoms) using the DBSCAN algorithm based on the atom–atom distance standard-deviation matrix. Two atoms become members of the same cluster if there is a neighbor-atom path between them, in which the distance between two neighboring atoms does not vary by a large amount. Also, atoms pairs that do not have enough similarly moving neighbors cannot be members of the same cluster.

The 8wt, 8m1, 11wt, 11m1, 11m2, 11m3 and 11m4 complexes were segmented into six, six, six, eight, three, eight and six parts, respectively (not counting the “non-segmented atoms” part). This highlights the 11m2 complex again as a complex with outlying dynamics. The segmentation algorithm also separates the conceptually distinct regions within the complex. Here the most significant of these were found to be the α_1_-helices of the inhibitor peptides, the beta-hairpins of mature myostatin and the myostatin main bodies (see Figure 11A). This way, we were able to distinguish the following different states of the system: in some complexes the two myostatin main bodies move together, and in some they move independently. The beta-hairpin can also move independently from the main bodies of is concert with them, or the beta-hairpin can fluctuate coupled to the inhibitor helix or separately. We always observed the helices to be in different segments from each other and from the non-hairpin part of the myostatin main body (Figure 11).

In the 8wt complex, the two myostatin main bodies become segmented together, but the hairpins form different independent segments (Figure 11B). So, it seems that in the complex formed with the natural α_1_-helix the β-hairpin “fingers” can move—as a rigid body—away from the main body of myostatin, possibly opening an exit route for the ligand. The hairpins probably function as controlled gates for the peptides, and the nature of their motion greatly influences peptide release. This mechanism, i.e., the control over the mobility and conformation of the hairpin, may help the wild-type myostatin and lasso-peptide to separate after the final proteolytic cleavage. In the 8m1 complex the correlation of the movement of the two main-bodies of myostatin vanishes, causing them to become different segments. Although both 8wt and 8m1 segment into six different parts, some parts in 8wt only contain a few residues, which also points to a more rigid complex in its case. The N-terminal part of one of the helices in 8m1 is partially unfolded, which is reflected by the non-segmented nature of these Cα atoms, resulting in elevated mobility in the peptides too. In the 11wt complex, the two non-hairpin parts of the myostatin are still independently segmented, but one of the beta-hairpins belongs to the same group as the proximal helix. We attributed this behavior to the presence of the E50–R333 interaction and the S55–V316 interaction, which anchor the hairpin to the helix. In the 11m1 complex, the full myostatin breaks approximately into two independent segments, due to the partial release of the hairpins upon the E50Y mutation. The full release happens when the S55W mutation is introduced in 11m2, which results in a fully unique segmentation pattern, namely a segment including the full myostatin—including the hairpins and the two helices representing two different groups. This, however, results in a rigid system where the gates are shut and bolted, leading to an efficiently inhibited state which not only locks the 11m2 peptides within the binding site but also obstructs the approach of the substrate helices. The L61I mutation of 11m3—such a small change—results in a segmentation pattern similar to that of the 8m1 complex, falling back to the more dynamic case and suspected easier release of the peptides. Similar to the case of 11wt, one of the hairpins sticks to the close helix, but in contrast, the two non-hairpin parts of the myostatin merge into one segment. This highlights the importance of the simultaneous presence of these releasing mutations, along with their synergetic effect on helix-hairpin binding.

## 3. Materials and Methods

### 3.1. Molecular Modeling, Dynamics Calculations and Trajectory Analysis

All simulations were started from the 5NTU PDB file [13], which was processed using the Maestro software of the Schrödinger Suite (Schrödinger, LLC, New York, NY, USA, 2021). First, the G269–S375 peptide-chain interval was extracted from chain A and duplicated. The duplicate chain was aligned to the corresponding interval in chain B. These two chains comprise the full myostatin prodomain. Then, the W44–L66 peptide-chain interval was extracted from chain B and duplicated, and the duplicate chain was aligned to the corresponding interval in chain A. These two chains comprise two 23-residue-long peptides. Mutations were introduced to create the 8wt, 8m1, 11wt, 11m1, 11m2, 11m3 and 11m4 peptides with PyMol. A C-terminal amide protection was also introduced for each peptide. As for the lasso-peptide (comprising 51 residues), the W44–Y94 interval was used for the simulations.

MD simulations were carried out by using the GROMACS 2021.4 software [45]. Complexes and peptides were solvated with an OPC water model [46], while sodium and chloride ions were added to electrostatically neutralize the system (final concentration of NaCl is 0.15 M). For all runs, the AMBER-ff99SBildnp-star [47] force field was used. To equilibrate the proteins, the following steps were employed: first, a steepest descent integrator with position restraints of 1000, 500, 100 and then 0 kJ × mol^−1^ × nm^−2^ and a maximal force tolerance of 50 kJ × mol^−1^ × nm^−2^ was used. Next, an NVT equilibration was conducted using the leap-frog integrator for 50,000 steps with a 2 fs step-size with position restraints of 1000, 500, 100 and 0 kJ × mol^−1^ × nm^−2^ at 310 K. Finally, an unconstrained NPT step was followed to allow introduction of pressure. Complexes and peptides were simulated for a total of 1 μs.

After the centering of the trajectories and the removal of water molecules and ions, the resulting trajectories were analyzed using the *gmx rama* command. In the case of the complex trajectories, a peptide-only trajectory was constructed, which contained only one of the two peptides in the complex. After the *gmx rama* command, all the Φ and Ψ dihedral-angles were plotted using the matplotlib Python package [48]. Also, for each dihedral angle pair a *d* Mahalanobis distance value was calculated using μ = [−70.79, −32.85] and *C* = [[300.13, −190.40], [−190.40, 362.08]]. Angles were measured in degrees. Then, all the exp(−*d*^2^) values were averaged for a run, resulting in the GMM score (reported as percentage values). The μ and *C* parameters come from the fitting of a 2D Gaussian distribution on the right-handed α-helix part of an aggregated Ramachandran plot, collected from several experimental protein structures (PDB files filtered by the PISCES server [49] with a maximum of 25% sequence identity, 2 Å resolution, 0.25 R-value and a protein length of 300).

For the opening angle and inter-domain dihedral angle calculations, the *gmx gangle* command was used. Trajectory clustering was achieved using the *gmx cluster* command with the “gromos” method, with a cutoff of 1.5 Å, and fitting was performed on the MainChain+Cβ atom set. H-bond data were obtained from a *gmx hbond* run with a hydrogen-donor–acceptor cutoff angle of 50° and a hydrogen-acceptor cutoff distance of 2.6 Å (i.e., with the *noda* and *nomerge* flags). Angle and H-bond distribution histograms were also created with matplotlib.

Secondary chemical interactions inside the complexes were determined by RING 3.0 with the default threshold options. Frames were analyzed with 400 ps time-differences which resulted in a total of 1001 frames. Then, in-house Python scripts were used to count the frame-averaged number of interactions. Interaction graph visualizations were created from these datasets using PyGraphViz 1.11 [50].

For the rigid body segmentation, a distance threshold (“eps”) of 2 Å and a neighbor count (“min_samples”) of 5 were used in the DBSCAN clustering algorithm. All visual inspections concerning residue–residue interactions were performed on the mid-structure of the most populated cluster of the ensembles.

### 3.2. Solid Phase Peptide Synthesis

The synthesis was performed on an HPPS-4000 apparatus (METALON Ltd., Budapest, Hungary) consisting of a Jasco LC-4000 series HPLC system, except without a PU-4180 HPLC pump, modified with an additional valve, allowing for recirculation and regulation of solvent flow [32]. ChromNAV2 software and the autosampler ensure the automated process. The PEEK chromatography column used as a fixed bed reactor tube for the resin and Dimethylformamide (DMF) was used as a solvent. A total of 150 mg of Fmoc-Rink amide Tentagel S RAM (0.24 mmol/g) resin (Iris Biotech GmbH) was used for the reactions. In the vials, 0.12 M of protected amino acids and Oxyma as the coupling reagent were dissolved in N-Methyl-Pyrrolidon (NMP) and injected into the resin-filled column. Couplings were performed in N,N-Diisopropylcarbodiimide (DIC), and for Fmoc-deprotection the cleavage solution consisted of 20 *v*/*v*% piperidine in DMF. During the synthesis, the pressure varied between 70 and 90 bar. The protocol for the synthesis steps is the following: flow: 0.3 mL/min (0–8 min); flow: 0.3 to 1.0 mL/min (8–9 min); flow: 1 mL/min (9–11.5 min) and flow 1.0 mL/min to 0.3 mL/min (11.5–12.0 min) [31].

### 3.3. Purification by High Liquid Performance Chromatography

The crude peptides were analyzed by reverse-phase HPLC on an analytical C-18 column (Phenomenex, Jupiter 5 μM, 250 × 4.6 mm, 100 Å) using gradient elution consisting of 0.1% TFA in water (eluent A) and 0.1% TFA acetonitrile/water 80/20 *v*/*v* (eluent B). The gradient was 0% to 70% B eluent in 70 min; the flow rate was 1 mL/min; and detection was made at 220 nm and 280 nm.

After the crude extract was analyzed, it was purified by RP-HPLC on a Kinetex 5u Evo C-18 column 100 A (150 × 21.2 mm) (Phenomenex, Torrance, CA, USA), using a gradient of water/acetonitrile. The solvent system was the following: 0.1% TFA in water and 0.1% TFA and 80% acetonitrile in water. The gradient was as 0 → 80% B in 80 min and flow 10 mL/min, and detection was made at 220 nm. Collected fractions were lyophilized.

### 3.4. Mass Spectrometry Analysis

Mass spectra were acquired with a Bruker AmaZon SL quadrupole ion trap instrument (Bruker Daltonics GmbH & Co. KG, Bremen, Germany) equipped with an electrospray (ES) source. Mass spectra were acquired in positive mode in the range *m*/*z* 50–1800. Samples were dissolved in a solvent mixture of acetonitrile-water (1:1, *v*/*v*) containing 0.1 *v*/*v*% formic acid. Sample solutions were introduced to the source by flow injection at a flow rate of 50 uL/min. Nitrogen was used as nebulizer gas and dry gas as well. Data were analyzed by the software Bruker DataAnalysis 4.1.

### 3.5. Circular Dichroism Measurements

FUV-ECD spectra were recorded on a Jasco J810 spectrophotometer (Jasco Corporation, Tokyo, Japan) by using a 1.0 mm path length cuvette with protein concentrations of 30–60 μM. Data accumulation was performed over a range of 185–260 nm, with 0.2 nm step resolution at a scan rate of 50 nm × min^−1^ with a 1 nm bandwidth. The spectral accumulations were resolved at 5, 15 and 25 °C, and the temperature dependent measurement in the case of the 11wt peptide was performed between 5 and 90 °C. The temperature was controlled by using a Peltier-type heating system. Each spectrum baseline was processed by subtracting the solvent spectrum from that of the protein and corrected by the cuvette size and the concentration of each of the samples and the raw ellipticity units [Θ]_MR_. In addition, the measured values were uniformly scaled to have a unit absolute mean using the methods described in the references [35,36].

### 3.6. CCA+ Calculations

In the first case, all the recorded spectra from FUV-ECD measurements included the temperature dependence and TFE dependence measurement and were deconvoluted using the CCA+ protocol as a mixture of unfolded (component 1) and folded (component 2) forms. Data can be seen in the results in the Supplementary Information Table S1 [37].

## 4. Conclusions

One possible strategy for the treatment of skeletal muscle-related disorders is the selective inhibition of myostatin (GDF8). Using ECD spectroscopy and MD simulations, we studied the ligand binding capacity of myostatin and the nature of the peptide segments that might be able to block its substrate-binding groove and thus become inhibitors of its function. We found that the sequence of the α_1_-helix of its pro-domain lasso-peptide (8wt) has been optimized to simultaneously fulfill two very different roles: to function as a strong binder as well as a good leaving group. We found that the 8wt peptide forms an outstanding number of non-covalent interactions with the mature core of myostatin and maintains the most helical, ordered conformation within the complex. We also found independent movement of the gate-keeper β-hairpin segment in the wild-type myostatin complex that assists the dissociation of the lasso-peptide. The 8wt sequence results in the least-ordered solvated form which provides extra stability for the dissociated state and discourages rebinding. The challenge of inhibitor design for such an intricately balanced and pliable system is that while the favorable interactions of the bound forms have to be maintained, the dissociation paths have to be effectively blocked. We found that the α_1_-helix of GDF11 that also evolved naturally to be the partner of a mature core nearly identical to that of myostatin is able to fulfill this purpose. The 11wt peptide is more helical in its stand-alone solvated state than 8wt, and we showed that a few well-placed mutations might further increase its helicity. The local, in-complex interactions can also be optimized to nearly mimic that of the natural variant. In fact, we identified a variant—11m2—that is sufficiently ordered both in solvent and within the inhibitory complex, forms a high number of contacts with the binding-pocket and induces such changes in its internal dynamics that lead to a rigidified, permanently locked conformation that traps the peptides in the binding sites. Based on these results, we propose that the GDF11 sequence could be used as scaffold when designing inhibitors of myostatin and emphasize the significance of not only aiming to optimize the pre-formed binding conformation by increasing helicity but also monitoring the changes that mutations introduce into the local interaction network and the overall flexibility of the inhibited complexes. The gate-like function of the β-hairpin and the opening/closing dynamics of the mature core structure of myostatin were first studied in detail in this work, suggesting a more complex build-up and regulation over the function of this vital regulator protein than previously considered.

## Figures and Tables

**Table 1:** The average number of the different interaction types between the different myostatin–peptide complexes.

	Average Number of the Different Interaction Types				
	Van der Waals	H-Bond	Ionic	Cation-π	π-π Stack
**8wt**	25.3	14.5	1.0	0.4	0.0
**8m1**	23.2	10.4	0.9	0.5	0.1
**11wt**	31.0	13.5	1.1	0.2	0.0
**11m1**	28.4	9.4	0.7	0.7	0.5
**11m2**	29.1	12.7	0.6	0.5	0.3
**11m3**	29.7	8.5	1.1	0.7	0.6
**11m4**	29.8	9.2	1.0	0.4	0.4

## Data Availability

Data is contained within the article or supplementary material.

## Supplementary Materials

The following supporting information can be downloaded at: https://www.mdpi.com/article/10.3390/molecules28155655/s1, Figure S1. Mass spectrum of peptide 8wt: *m*/*z* calculated for the peptide [M]+ 2881.3, found 2880.8. Figure S2. Mass spectrum of peptide 8m1: *m*/*z* calculated for the peptide [M]+ 2797.3, found 2796.7. Figure S3. Mass spectrum of peptide 11wt: *m*/*z* calculated for the peptide [M]+ 2876.4, found 2875.7. Figure S4. Mass spectrum of peptide 11m1: *m*/*z* calculated for the peptide [M]+ 2909.7, found 2910.4. Figure S5. Mass spectrum of peptide 11m2: *m*/*z* calculated for the peptide [M]+ 3009.6, found 3008.8. Figure S6. Mass spectrum of peptide 11m3: *m*/*z* calculated for the peptide [M]+ 3009.6, found 3008.8. Figure S7. Mass spectrum of peptide 11m4: *m*/*z* calculated for the peptide [M]+ 2975.5, found 2975.6. Figure S8. Mass spectrum of lasso 51 peptide: *m*/*z* calculated for the peptide [M]+ 5982.1, found 5980.6. Figure S9. FUV-ECD temperature dependence measurements at 5, 15 and 25 °C. Figure S10. FUV-ECD temperature dependence measurements at 5, 15 and 25 °C with 10% of TFE added to the samples. Figure S11. FUV-ECD temperature dependence measurements of the 11wt peptide at 5 and 90 °C. Figure S12. Ramachandran-plots of the ensembles of the α_1_ peptides from the MD simulation carried out in solvent using the physiological 0.15 M salt (NaCl) concentration. Figure S13. Ramachandran-plots of the ensembles of the α_1_ peptides from the MD simulation carried out in solvent using the physiological 0.15 M salt (NaCl) concentration. Figure S14. Ramachandran-plots of the simulated peptides in complex. Figure S15. The RING analysis map of the simulated peptide 8m1 in complex. Figure S16. The RING analysis map of the simulated peptide 11wt in complex. Figure S17. The RING analysis map of the simulated peptide 11m1 in complex. Figure S18. The RING analysis map of the simulated peptide 11m2 in complex. Figure S19. The RING analysis map of the simulated peptide 11m3 in complex. Figure S20. The RING analysis map of the simulated peptide 11m4 in complex. Figure S21. The RBS of the simulated peptides (8wt, 8m1, 11wt, 11m1, 11m2, 11m3, 11m4) in complex. Table S1. The ratios of the nonhelical (Component 1.) and helical (Component 2.) part of the peptides alone in different temperatures with and without 10% TFE content. The ratios from the measured ECD spectra were calculated by the CCA+ program. Table S2. Measured and calculated average helicity values of the α_1_ peptides–calculated values are derived from MD simulations carried out in 0.5 M NaCl and 0.5 M NaCl medium, using default helicity metric of GROMACS and by the GMM metric developed by us.

## Abbreviations

ALK4/5/7	Activin-like receptor Kinase receptor 4/5/7
BMP-1	Bone Morphogenetic Protein-1
CD	Circular Dichroism
DIC	N,N-Diisopropylcarbodiimide
DMF	Dimethylformamide
ECD	Electronic Circular Dichroism
ESI-ITMS	Electrospray Ionization Ion Trap Mass Spectrometry
FUV-ECD	Far-UV Electronic Circular Dichroism
GDF11	Growth Differentiation Factor 11
GDF8	Growth Differentiation Factor 8
GMM	Gaussian Mahalanobis Mean
GMX	GROMACS
MD	Molecular Dynamics
MS	Mass Spectrometry
NMP	N-Methyl-Pyrrolidon
PDB	Protein Data Bank
RP-HPLC	Reverse Phase—High Performance Liquid Chromatography
StD	Standard Deviation
TFA	Trifluoroacetic Acid
TFE	2,2,2-Trifluoroethanol
TGF-β	Transforming Growth Factor β
