# Supplementary material for: Inhibitor Design Strategy for Myostatin: Dynamics and Interaction Networks Define the Affinity and Release Mechanisms of the Inhibited Complexes
Source: Molecules. 2023 Jul 26;28(15):5655. doi: 10.3390/molecules28155655 (PMC10420283; doi:10.3390/molecules28155655)
Supplement: Supplementary file 1 [file molecules-28-05655-s001.zip › molecules-2509399-supplementary.pdf]

Supplementary information

Mass Spectrometry analysis:

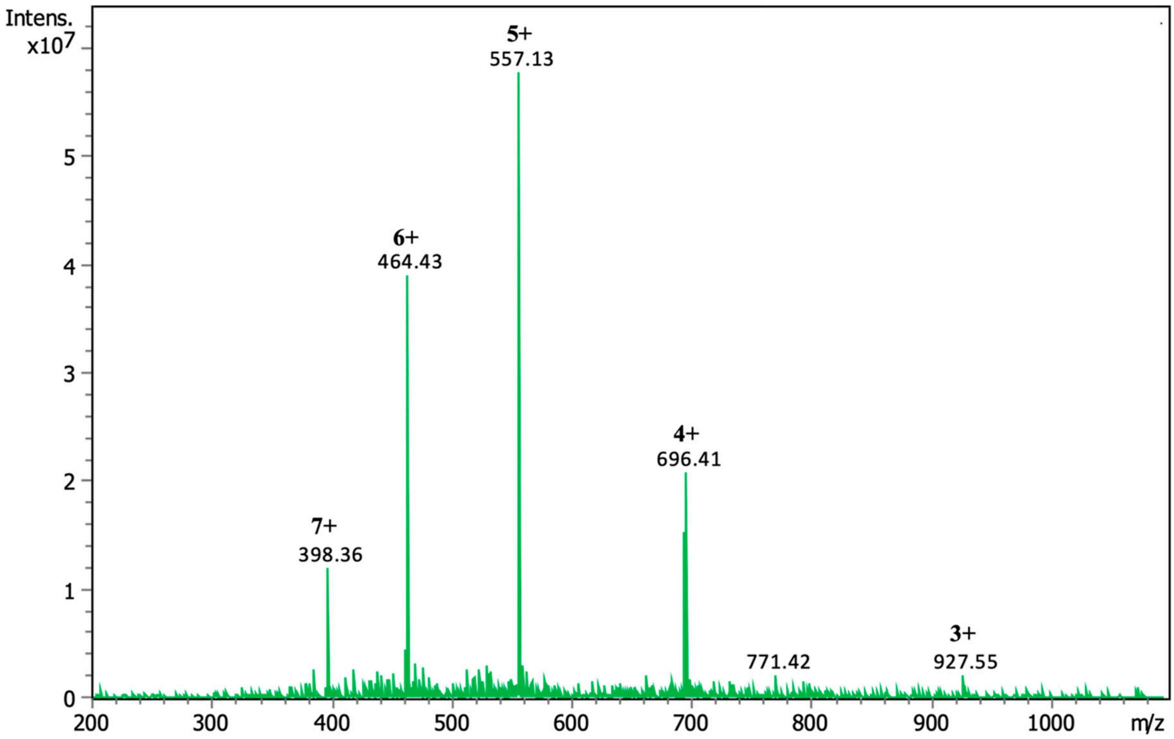

Figure S1. Mass spectrum of peptide 8wt: m/z calculated for the peptide $[M]^+$ 2881.3, found 2880.8.

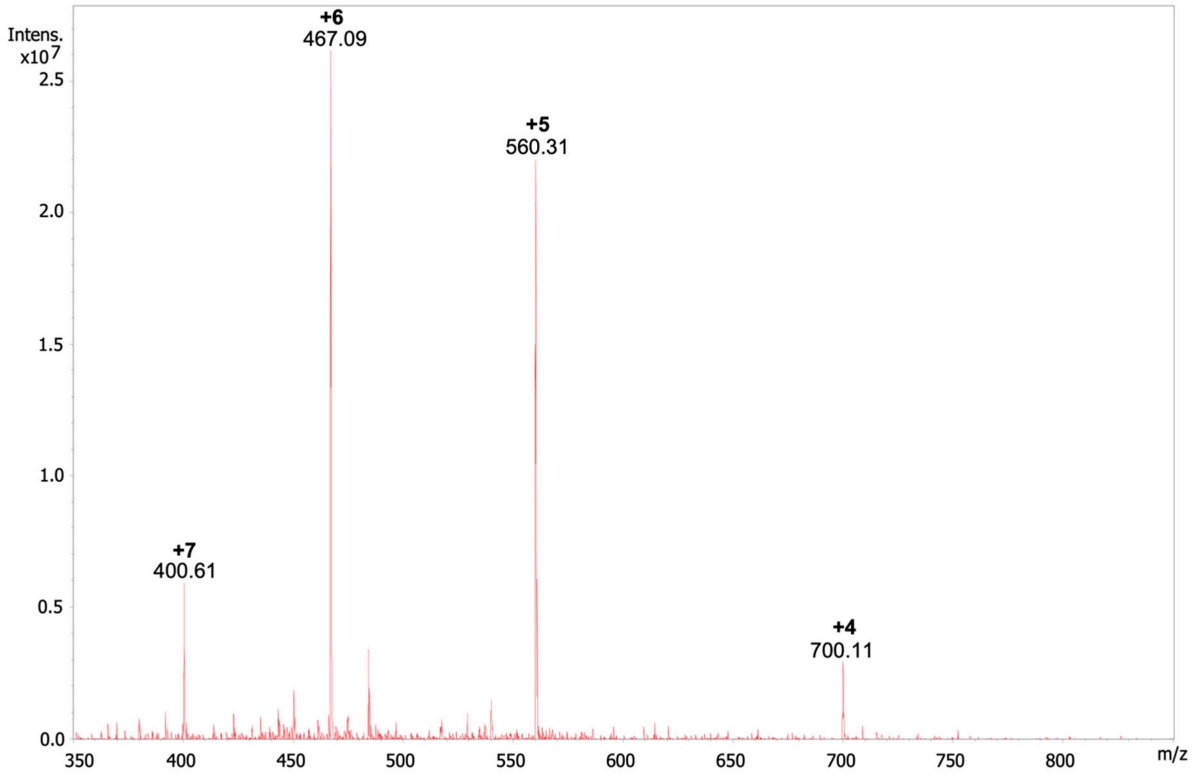

Figure S2. Mass spectrum of peptide 8m1: m/z calculated for the peptide $[M]^+$ 2797.3, found 2796.7.

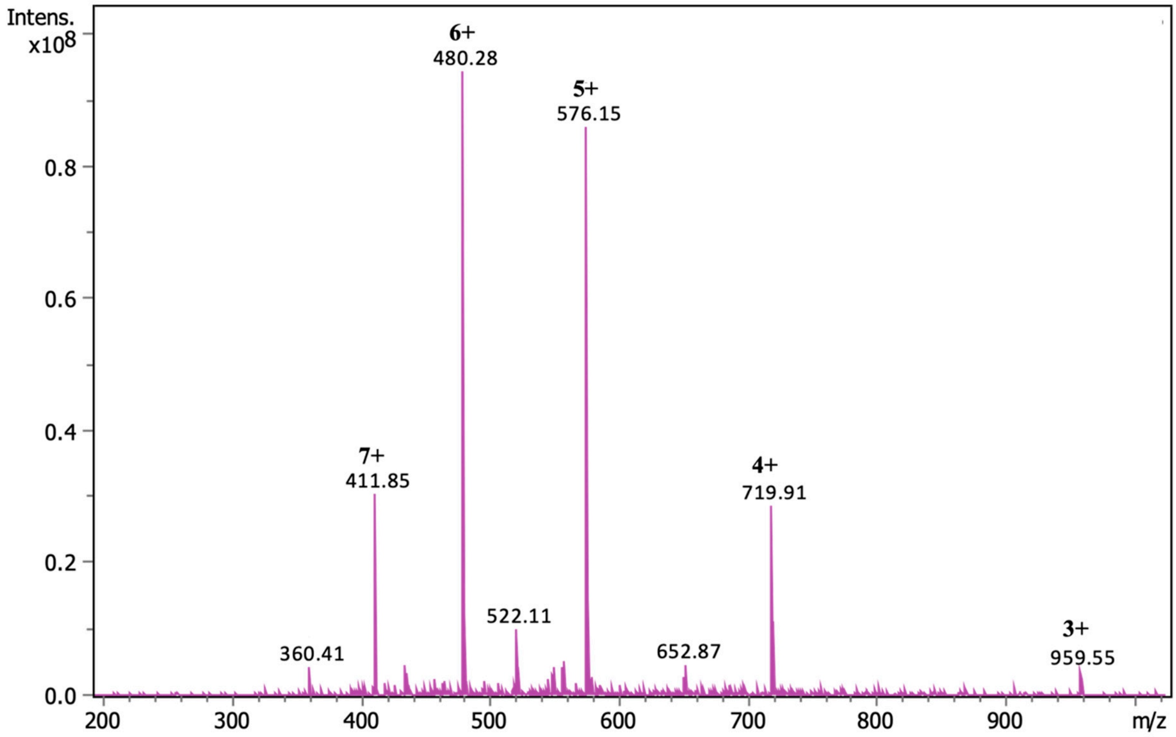

Figure S3. Mass spectrum of peptide 11wt: m/z calculated for the peptide $[M]^+$ 2876.4, found 2875.7.

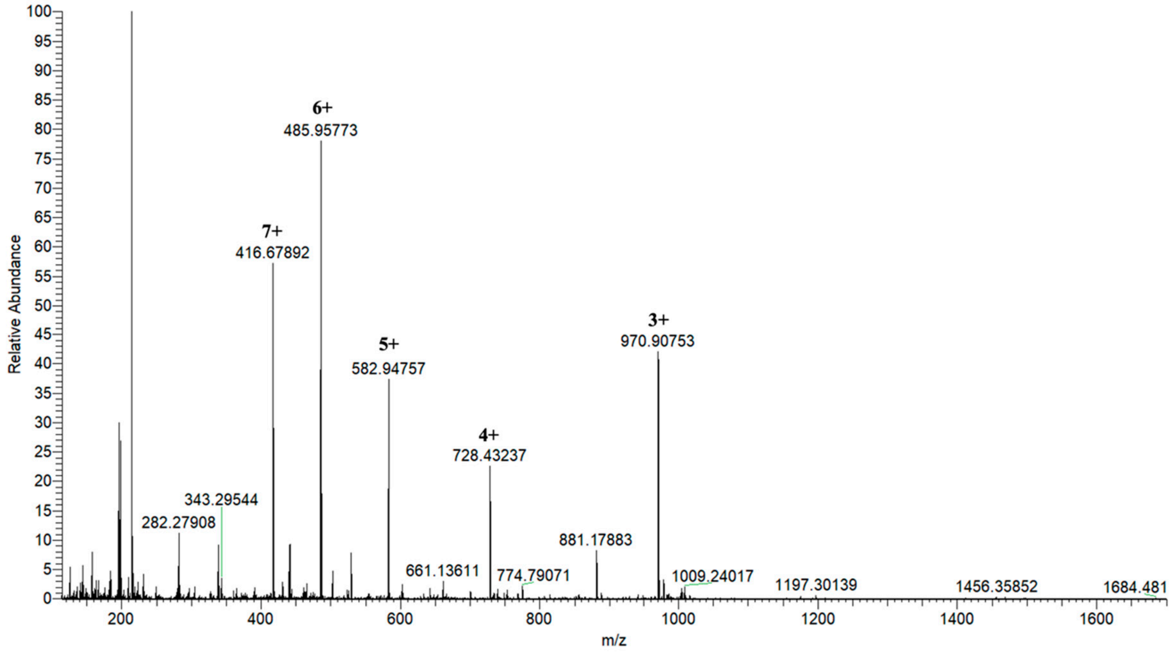

Figure S4. Mass spectrum of peptide 11m1: m/z calculated for the peptide $[M]^+$ 2909.7, found 2910.4.

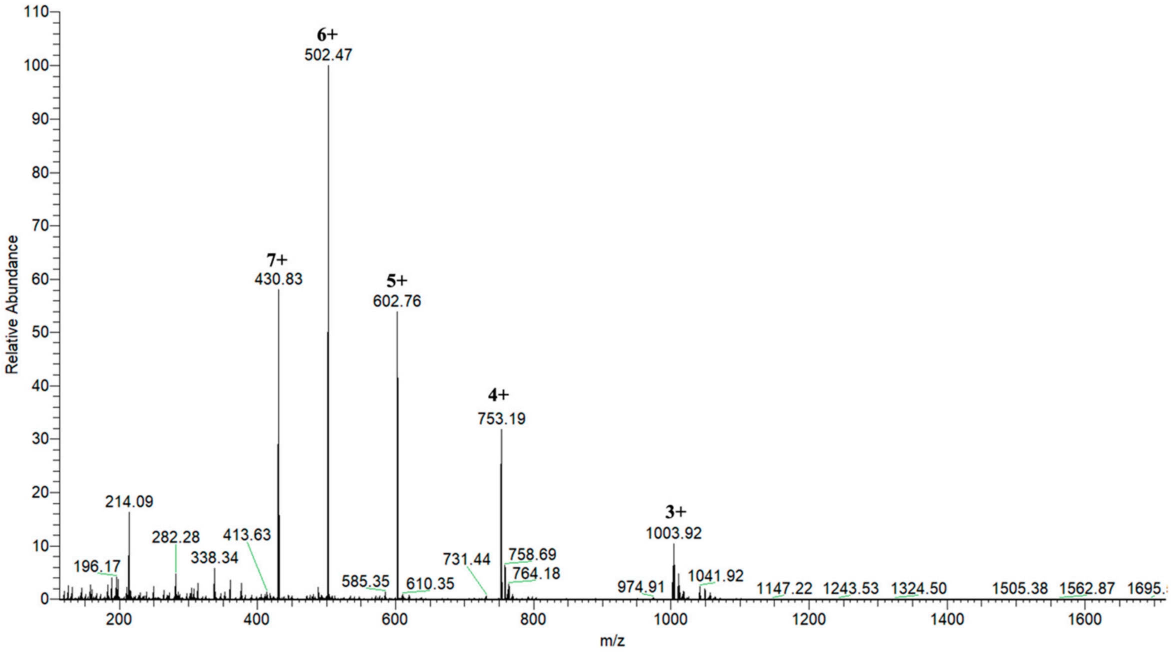

Figure S5. Mass spectrum of peptide 11m2: m/z calculated for the peptide $[M]^+$ 3009.6, found 3008.8.

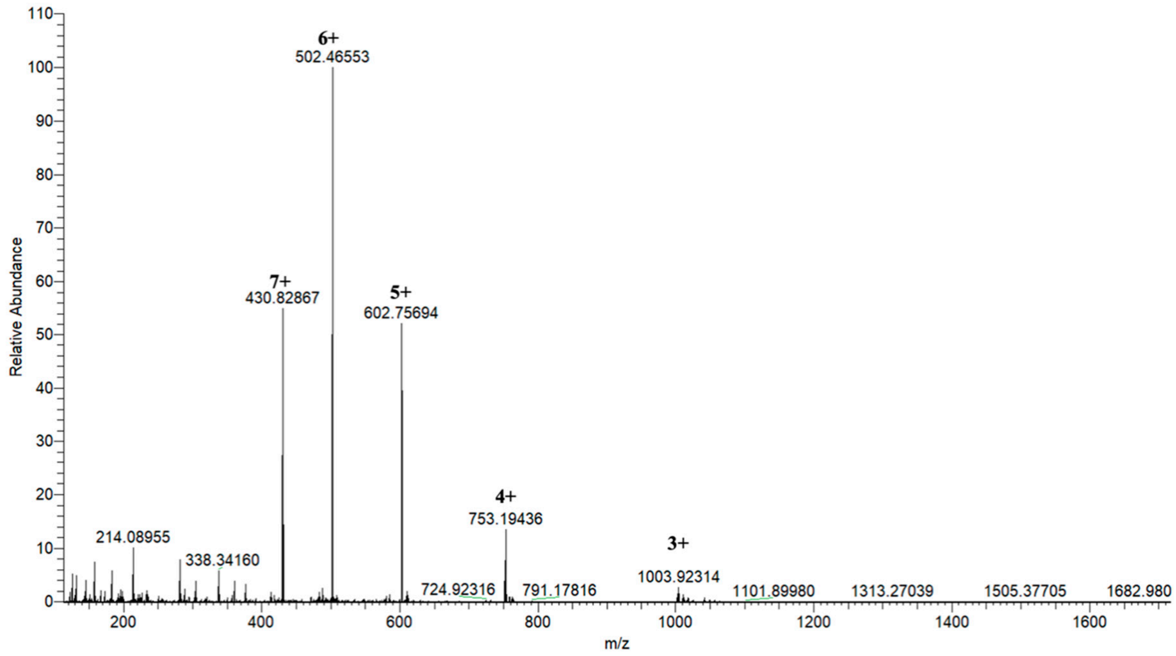

Figure S6. Mass spectrum of peptide 11m3: m/z calculated for the peptide $[M]^+$ 3009.6, found 3008.8.

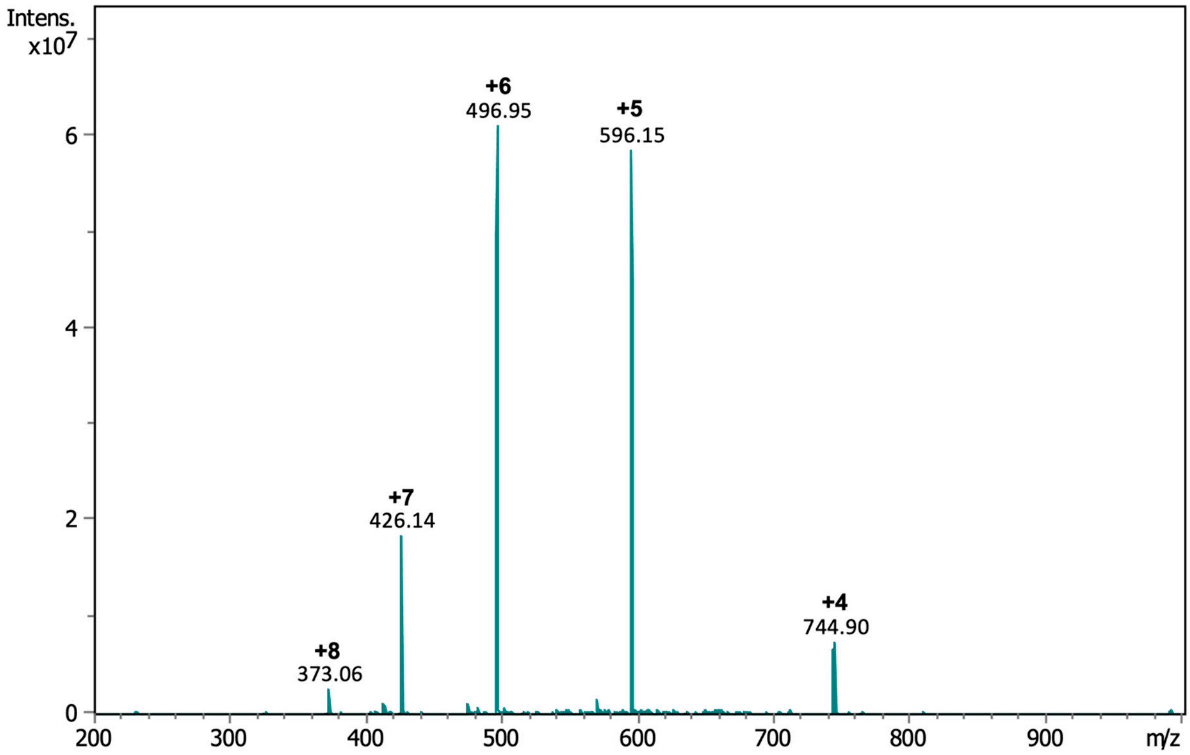

Figure S7. Mass spectrum of peptide 11m4: m/z calculated for the peptide $[M]^+$ 2975.5, found 2975.6.

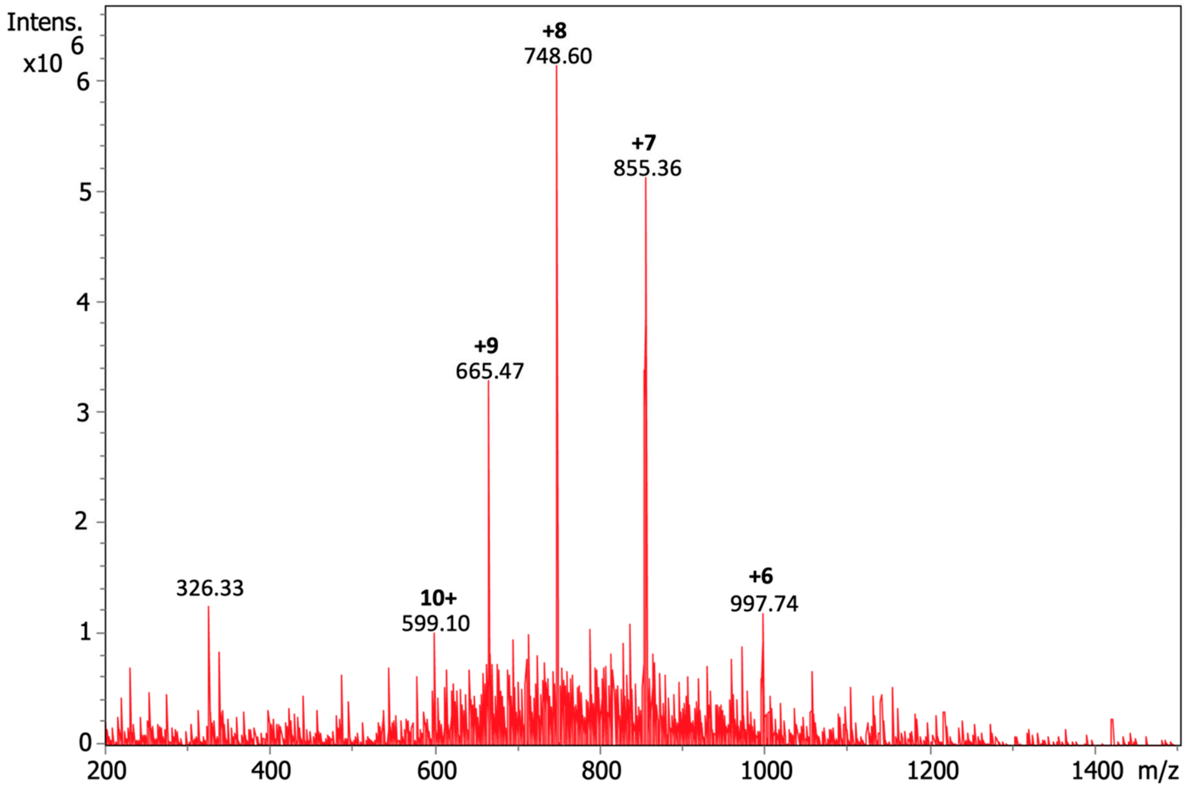

Figure S8. Mass spectrum of lasso 51 peptide: m/z calculated for the peptide $[M]^+$ 5982.1, found 5980.6.

Electronic Circular Dichroism measurement:

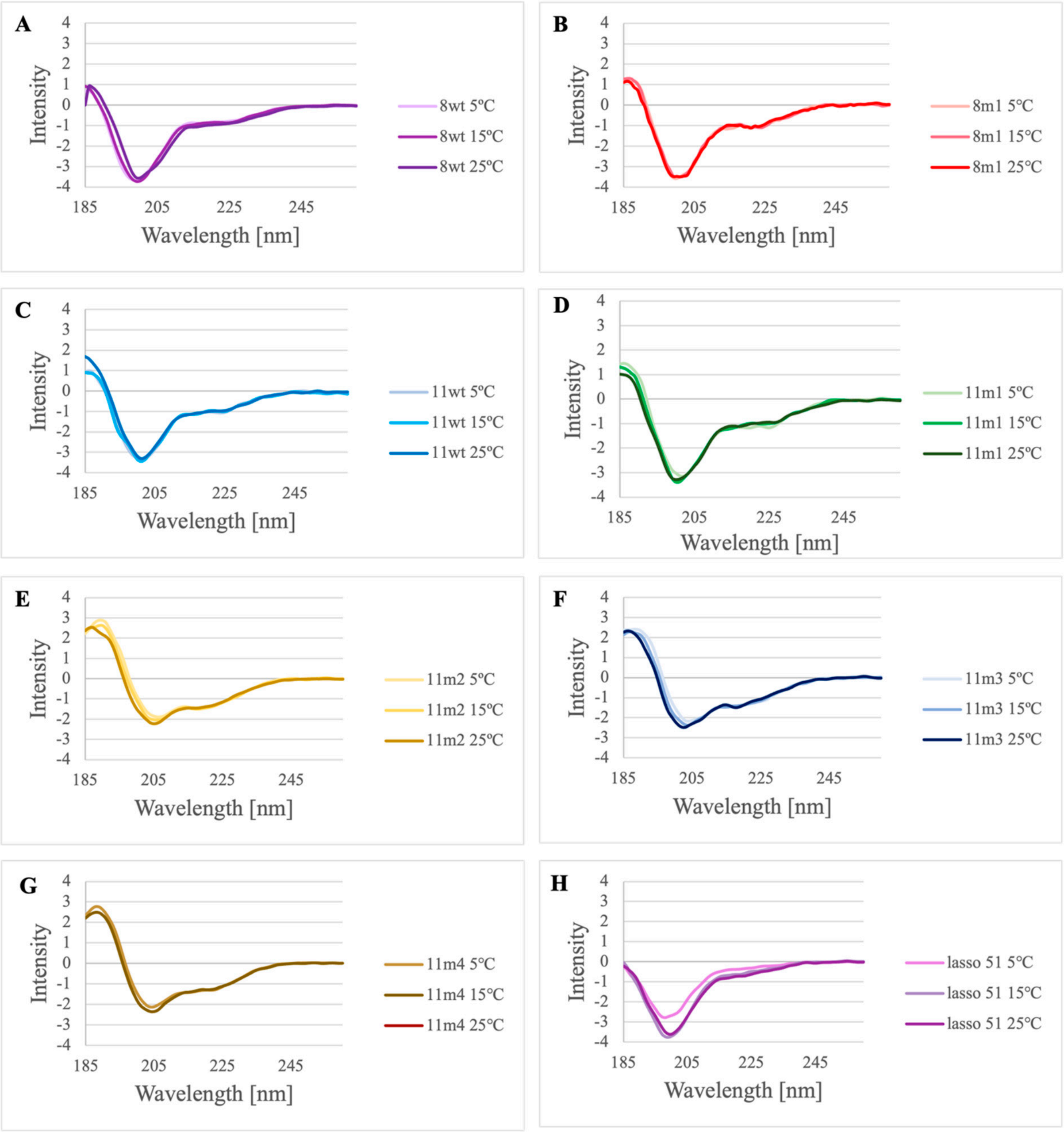

Figure S9. FUV-ECD temperature dependence measurements at 5, 15 and 25˚C. **A:** Measurement of the peptide 8wt. **B:** Measurement of the peptide 8m1. **C:** Measurement of the peptide 11wt. **D:** Measurement of the peptide 11m1. **E:** Measurement of the peptide 11m2. **F:** Measurement of the peptide 11m3. **G:** Measurement of the peptide 11m4. **H:** Measurement of the lasso 51 peptide.

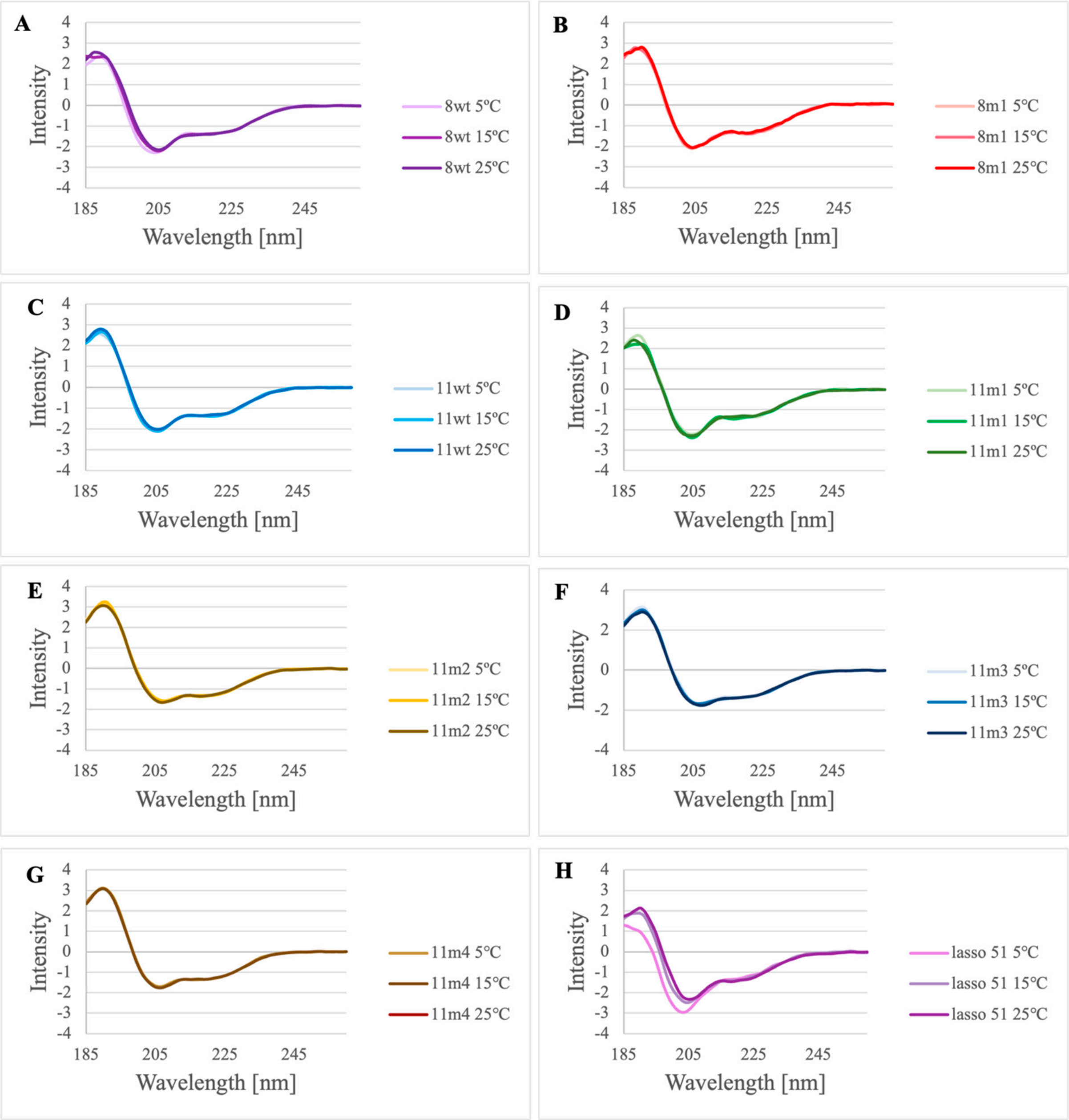

Figure S10. FUV-ECD temperature dependence measurements at 5, 15 and 25˚C with 10 % of TFE added to the samples. **A:** Measurement of the peptide 8wt. **B:** Measurement of the peptide 8m1. **C:** Measurement of the peptide 11wt. **D:** Measurement of the peptide 11m1. **E:** Measurement of the peptide 11m2. **F:** Measurement of the peptide 11m3. **G:** Measurement of the peptide 11m4. **H:** Measurement of the lasso 51 peptide.

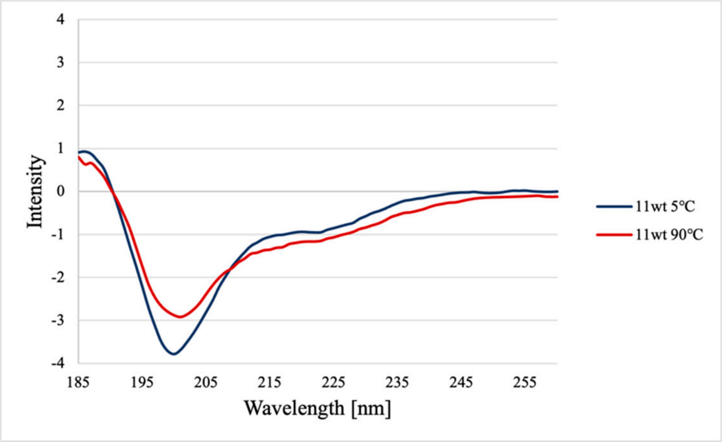

Figure S11. FUV-ECD temperature dependence measurements of the 11wt peptide at 5 and 90˚C.

Table S1. The ratios of the nonhelical (Component 1.) and helical (Component 2.) part of the peptides alone in different temperatures with and without 10 % TFE content. The ratios from the measured ECD spectra were calculated by the CCA+ program.

	**Peptide helicity (%) without added TFE**								**Peptide helicity (%) with 10 % TFE added**					
	5˚C		15˚C		25˚C		90˚C		5˚C		15˚C		25˚C	
	Comp .1.	Comp. 2.	Comp. 1.	Comp. 2.	Comp. 1.	Comp. 2.	Comp. 1.	Comp. 2.	Com p. 1.	Comp. 2.	Comp. 1.	Comp. 2.	Comp. 1.	Com p. 2.
**8wt**	100.0	0.0	97.3	2.7	86.0	14.0	-	-	36.1	63.9	29.8	70.2	26.0	74.0
**8m1**	81.7	18.3	82.5	17.5	86.5	13.5	-	-	23.3	76.7	22.1	77.9	20.8	79.2
**11wt**	84.9	15.1	84.3	15.7	76.8	23.2	-	-	24.1	75.9	22.0	78.0	19.4	80.6
**11m1**	72.2	27.8	79.1	20.9	83.1	16.9	-	-	29.4	70.6	32.9	67.1	33.8	66.2
**11m2**	13.3	86.7	21.1	78.9	29.8	70.2	-	-	0.0	100.0	0.1	99.9	1.8	98.2
**11m3**	28.2	71.8	38.3	61.7	46.1	53.9	-	-	3.4	96.6	5.4	94.6	6.9	93.1
**11m4**	24.9	75.1	33.4	66.6	41.7	58.3	-	-	6.5	93.5	7.6	92.4	8.6	91.4
**11wt temp. depend.**	90.0	10.0	-	-	-	-	78.3	21.7	-	-	-	-	-	-

Molecular dynamics simulations:

Table S2. Measured and calculated average helicity values of the α_1 peptides – calculated values are derived from MD simulations carried out in 0.5 M NaCl and 0.5 M NaCl medium, using default helicity metric of GROMACS and by the GMM metric developed by us.

**Peptide helicity (%):**		**0.5 M NaCl**		**0.15 M NaCl**	
	ECD measuremen t (CCA+)	calculated helicity	calculated GMM score	calculated helicity	calculated GMM score
**8wt**	14.0	20.6	26.1	15.5	22.3
**8m1**	13.5	18.7	25.9	23.4	29.8
**11wt**	23.2	18.7	26.6	29.6	34.4
**11m1**	16.9	15.8	20.7	30.0	36.6
**11m2**	70.2	24.3	30.4	21.0	31.9
**11m3**	53.9	26.1	33.8	17.9	25.6
**11m4**	58.3	19.6	26.2	22.1	29.7

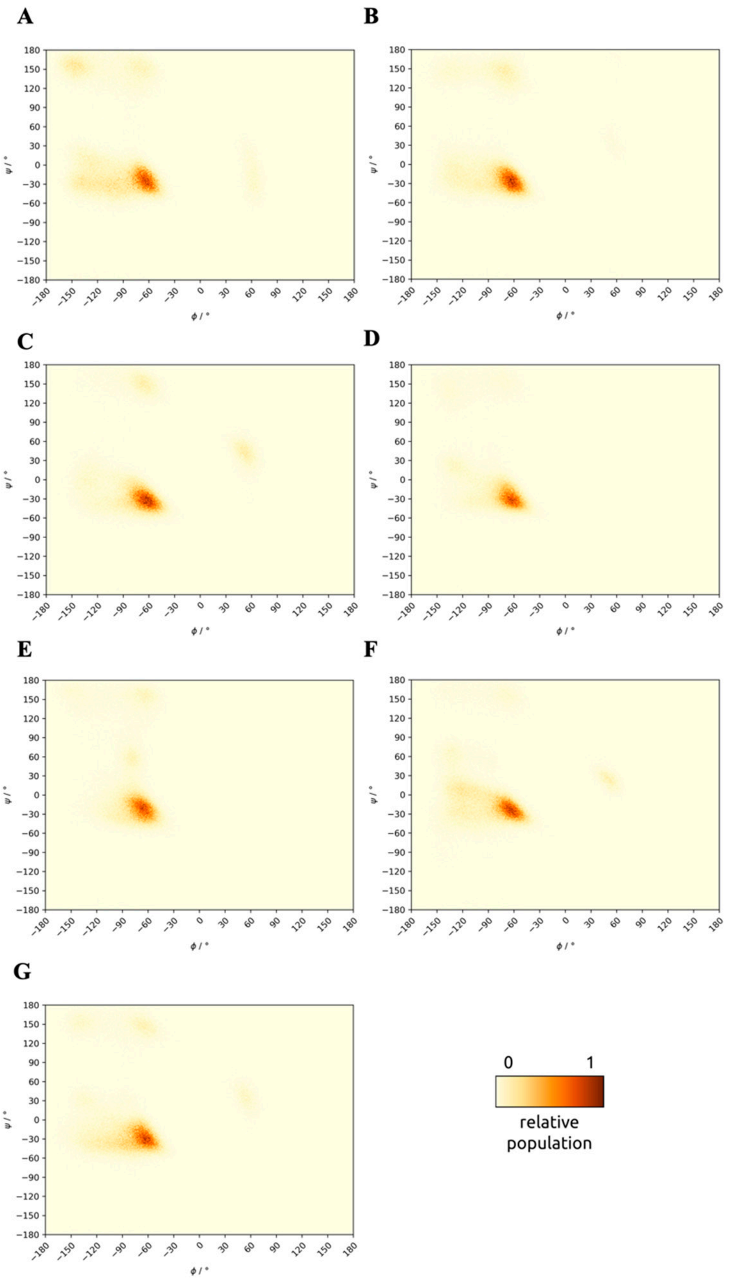

Figure S12. Ramachandran-plots of the ensembles of the α_1 peptides from the MD simulation carried out in solvent using the physiological 0.15 M salt (NaCl) concentration. The simulations were performed at a 0.15 M NaCl medium. **A.** Peptide 8wt. **B.** Peptide 8m1. **C.** Peptide 11wt. **D.** Peptide 11m1. **E.** Peptide 11m2. **F.** Peptide 11m3. **G.** Peptide 11m4.

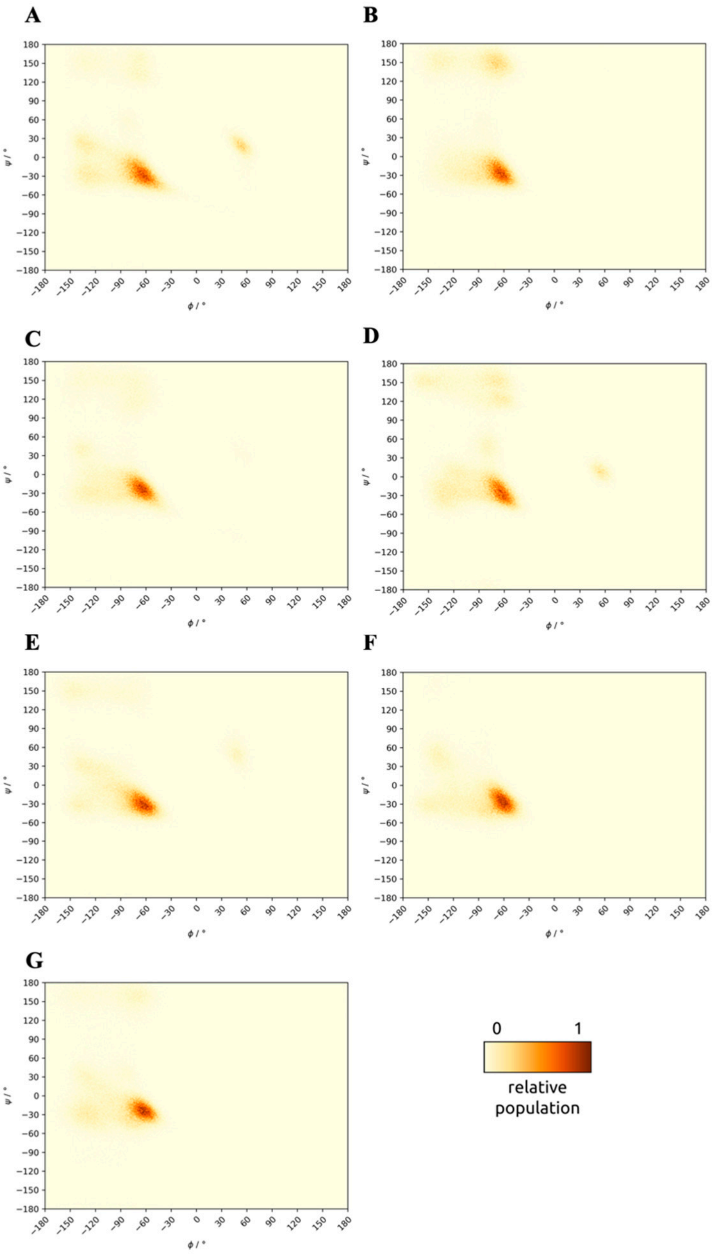

Figure S13. Ramachandran-plots of the ensembles of the α_1 peptides from the MD simulation carried out in solvent using the physiological 0.15 M salt (NaCl) concentration. The simulations were performed at a 0.5 M NaCl medium. **A.** Peptide 8wt. **B.** Peptide 8m1. **C.** Peptide 11wt. **D.** Peptide 11m1. **E.** Peptide 11m2. **F.** Peptide 11m3. **G.** Peptide 11m4.

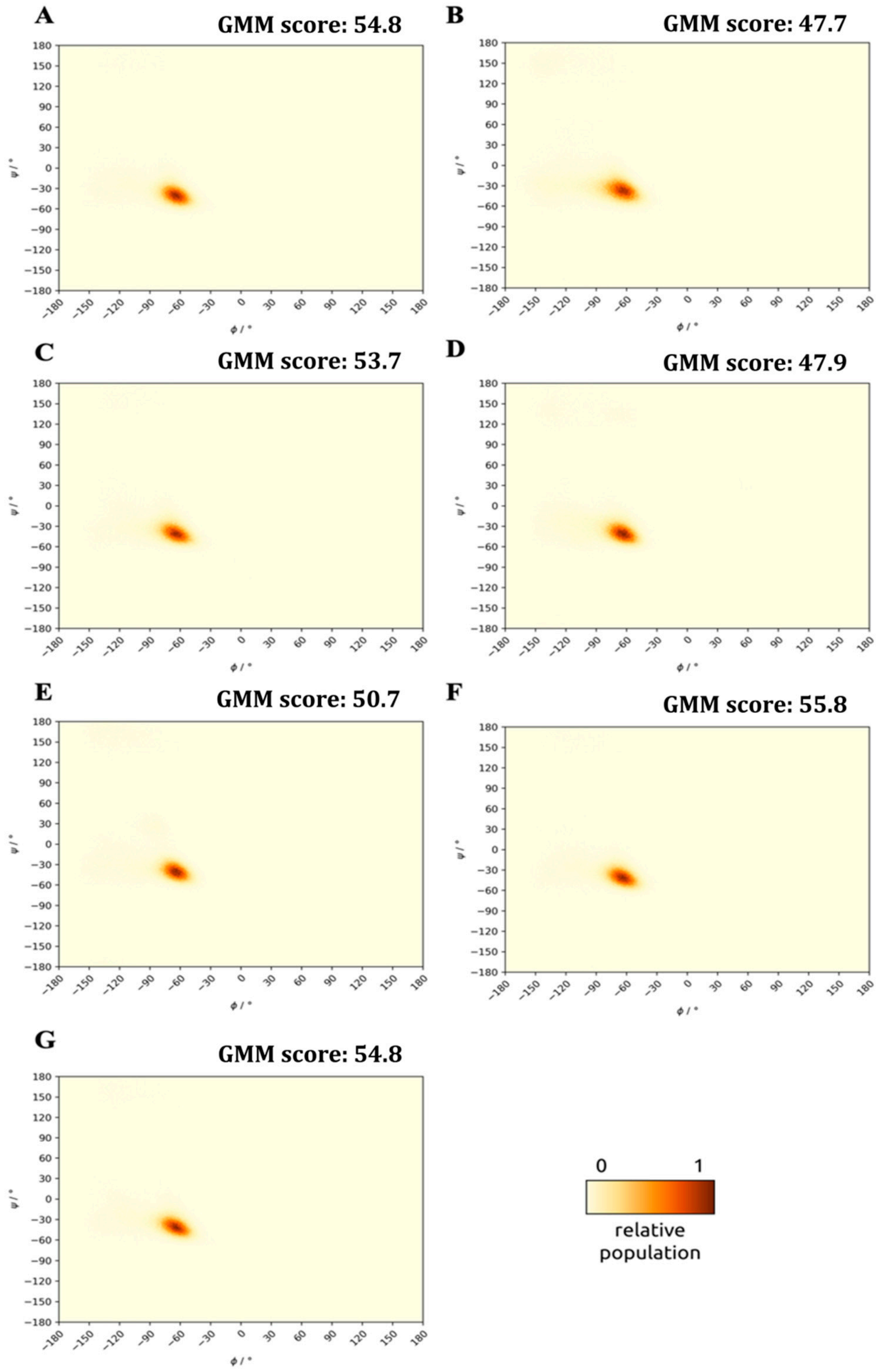

Figure S14. Ramachandran-plots of the simulated peptides in complex. The simulations were performed at a 0.15 M NaCl medium. **A.** Peptide 8wt in complex. **B.** Peptide 8m1 in complex. **C.** Peptide 11wt in complex. **D.** Peptide 11m1 in complex. **E.** Peptide 11m2 in complex. **F.** Peptide 11m3 in complex. **G.** Peptide 11m4 in complex.

Ring analysis:

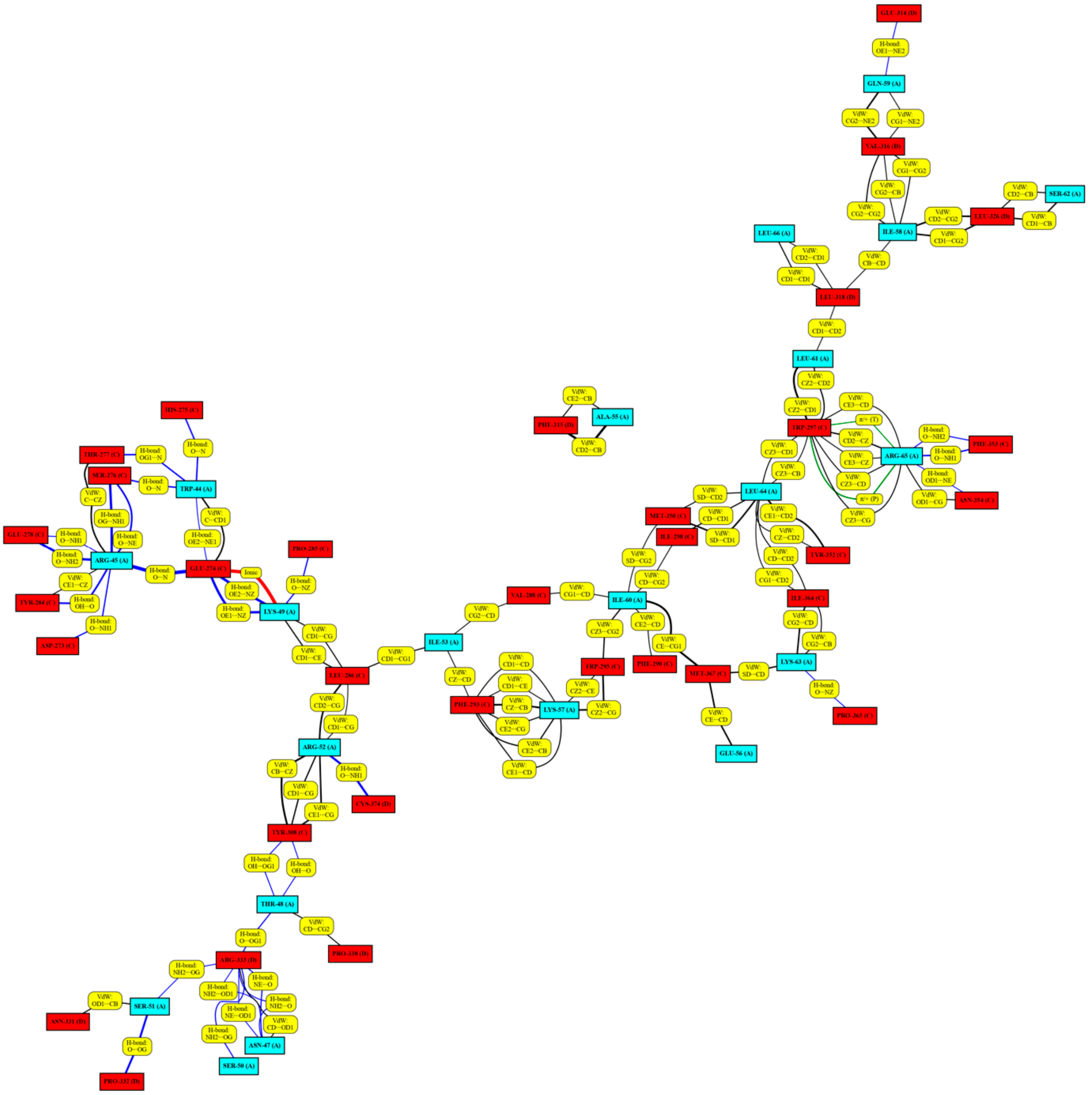

Figure S15. The RING analysis map of the simulated peptide 8m1 in complex. The amino acids in cyan boxes represents the α-helix peptide chain members, while the amino acids in red boxes represents the mature myostatin chain members interacting with the α-helix peptide chain members. Yellow boxes explain the different interaction types between the individual amino acid pairs. Lines of different thickness and color indicate the different interaction types between the individual amino acid pairs: **black**: Van der Waals interaction, **blue**: H-bond, **green**: Cation-π and π-π stack, **red**: ionic interaction. The thickness of the lines indicates the strength of the interaction, the thicker the line, the greater the percentage of the frames where the interaction is present.

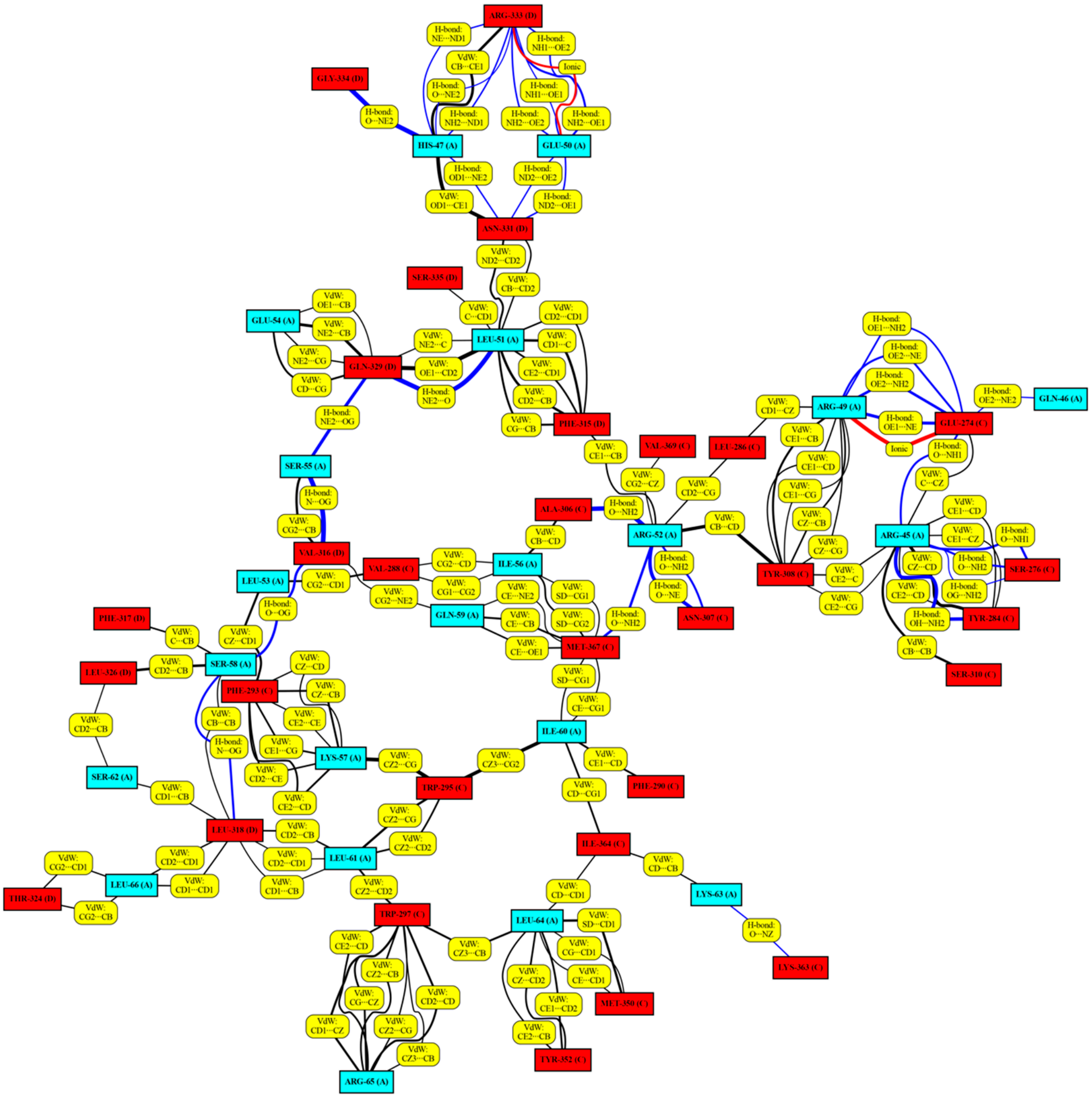

Figure S16. The RING analysis map of the simulated peptide 11wt in complex. The amino acids in cyan boxes represents the α-helix peptide chain members, while the amino acids in red boxes represents the mature myostatin chain members interacting with the α-helix peptide chain members. Yellow boxes explain the different interaction types between the individual amino acid pairs. Lines of different thickness and color indicate the different interaction types between the individual amino acid pairs: **black**: Van der Waals interaction, **blue**: H-bond, **red**: ionic interaction. The thickness of the lines indicates the strength of the interaction, the thicker the line, the greater the percentage of the frames where the interaction is present.

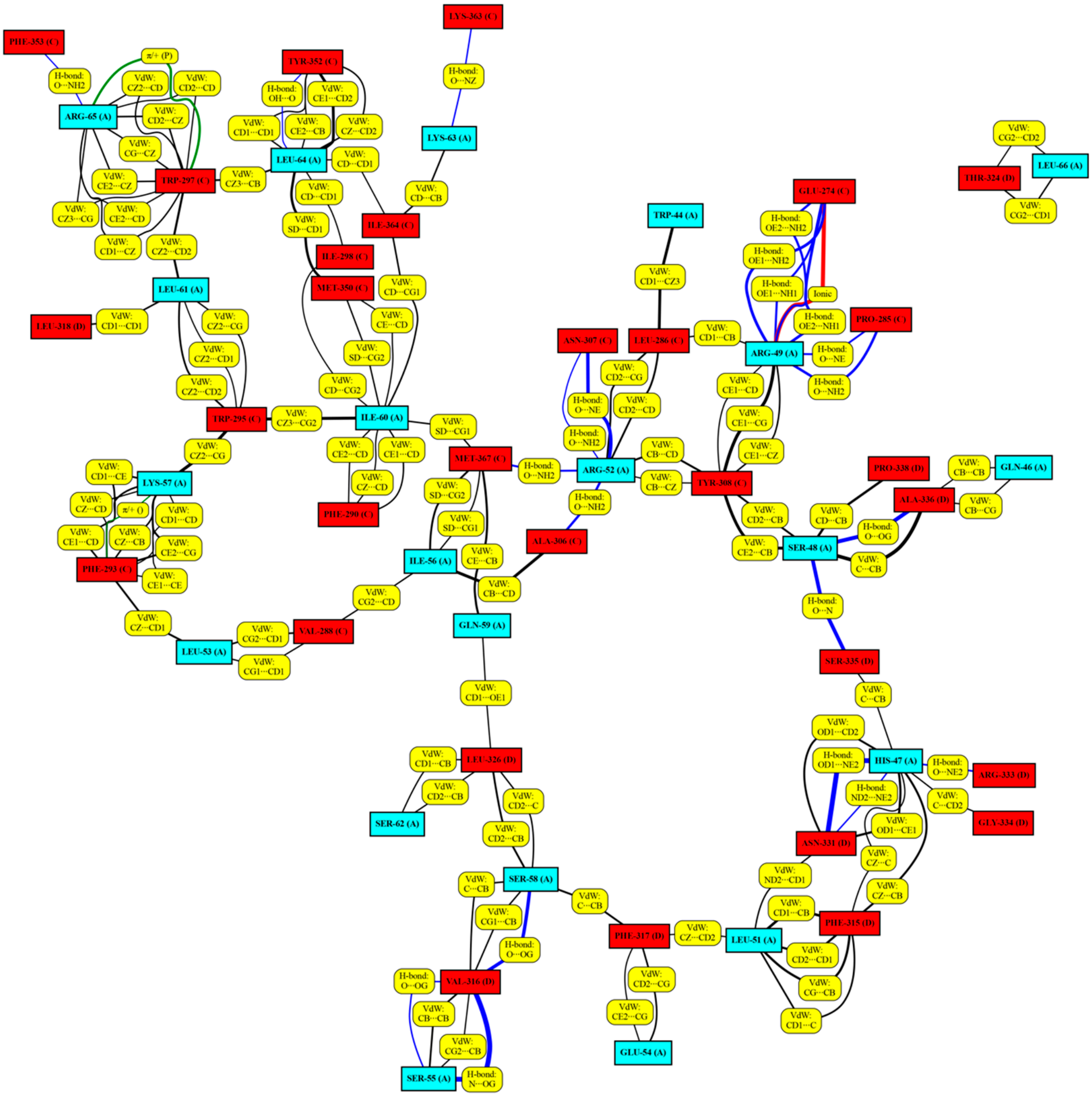

Figure S17. The RING analysis map of the simulated peptide 11m1 in complex. The amino acids in cyan boxes represents the α-helix peptide chain members, while the amino acids in red boxes represents the mature myostatin chain members interacting with the α-helix peptide chain members. Yellow boxes explain the different interaction types between the individual amino acid pairs. Lines of different thickness and color indicate the different interaction types between the individual amino acid pairs: **black**: Van der Waals interaction, **blue**: H-bond, **green**: Cation-π and π-π stack, **red**: ionic interaction. The thickness of the lines indicates the strength of the interaction, the thicker the line, the greater the percentage of the frames where the interaction is present.

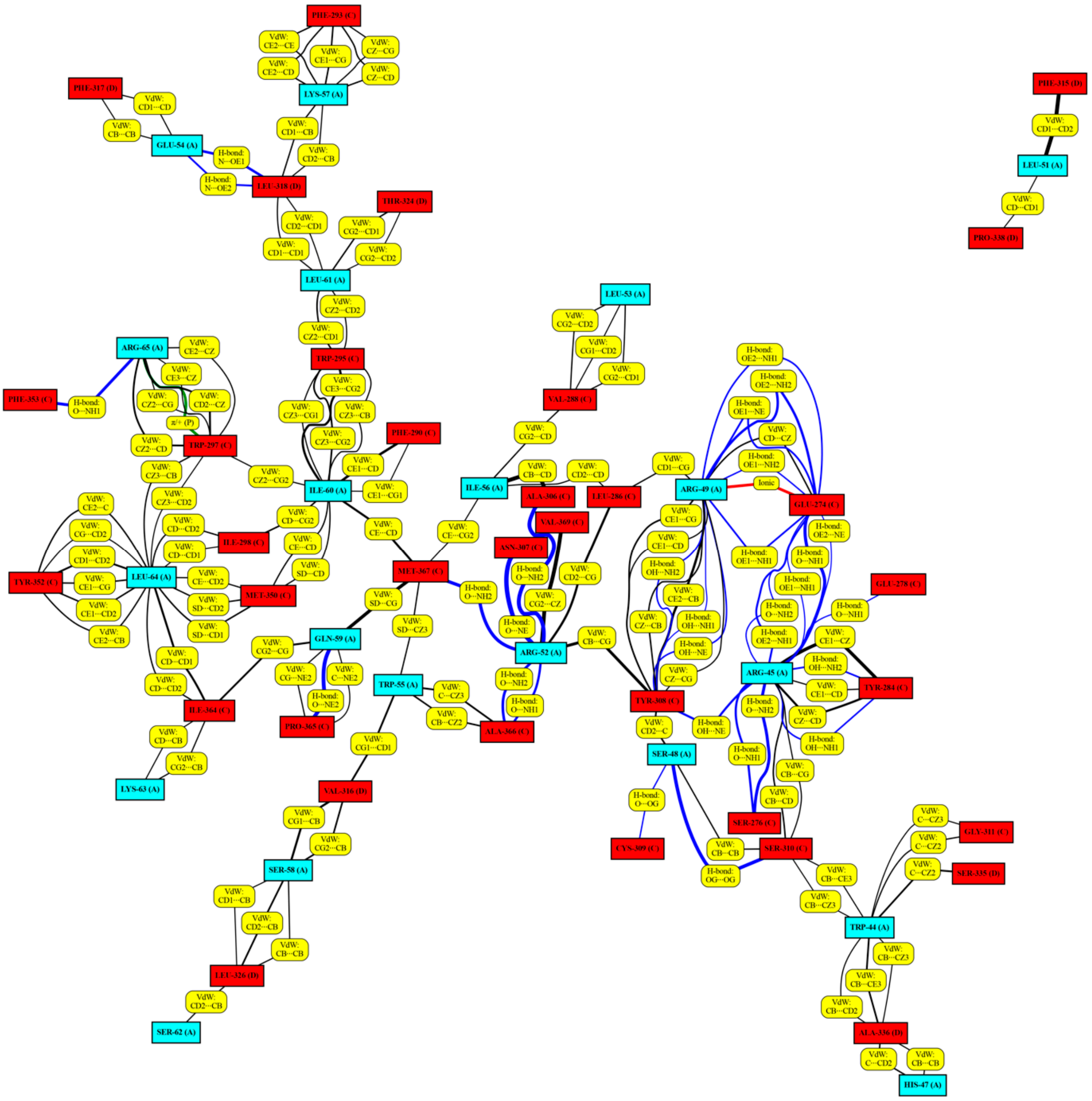

Figure S18. The RING analysis map of the simulated peptide 11m2 in complex. The amino acids in cyan boxes represents the α-helix peptide chain members, while the amino acids in red boxes represents the mature myostatin chain members interacting with the α-helix peptide chain members. Yellow boxes explain the different interaction types between the individual amino acid pairs. Lines of different thickness and color indicate the different interaction types between the individual amino acid pairs: **black**: Van der Waals interaction, **blue**: H-bond, **green**: Cation-π and π-π stack, **red**: ionic interaction. The thickness of the lines indicates the strength of the interaction, the thicker the line, the greater the percentage of the frames where the interaction is present.

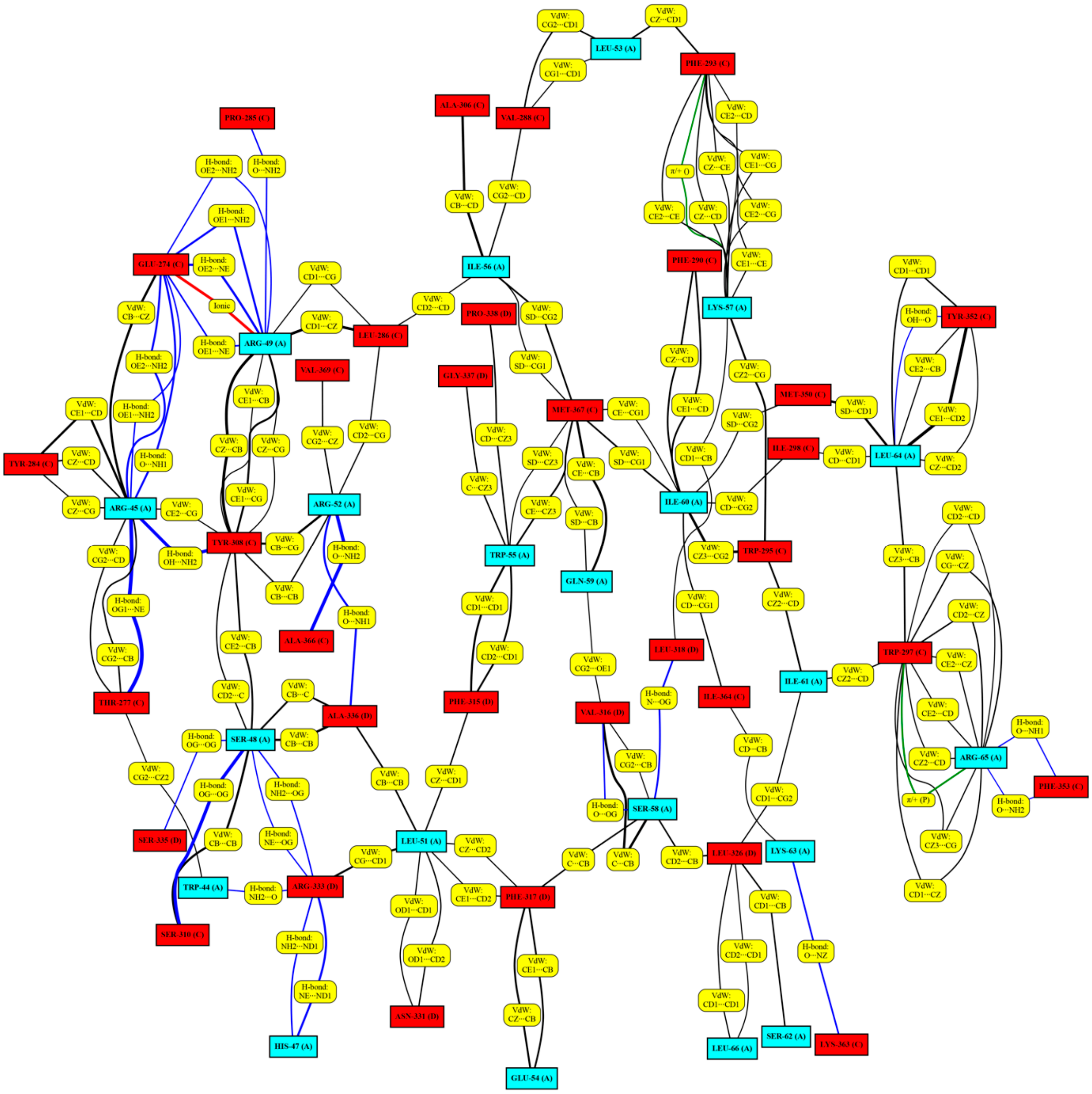

Figure S19. The RING analysis map of the simulated peptide 11m3 in complex. The amino acids in cyan boxes represents the α-helix peptide chain members, while the amino acids in red boxes represents the mature myostatin chain members interacting with the α-helix peptide chain members. Yellow boxes explain the different interaction types between the individual amino acid pairs. Lines of different thickness and color indicate the different interaction types between the individual amino acid pairs: **black**: Van der Waals interaction, **blue**: H-bond, **green**: Cation-π and π-π stack, **red**: ionic interaction. The thickness of the lines indicates the strength of the interaction, the thicker the line, the greater the percentage of the frames where the interaction is present.

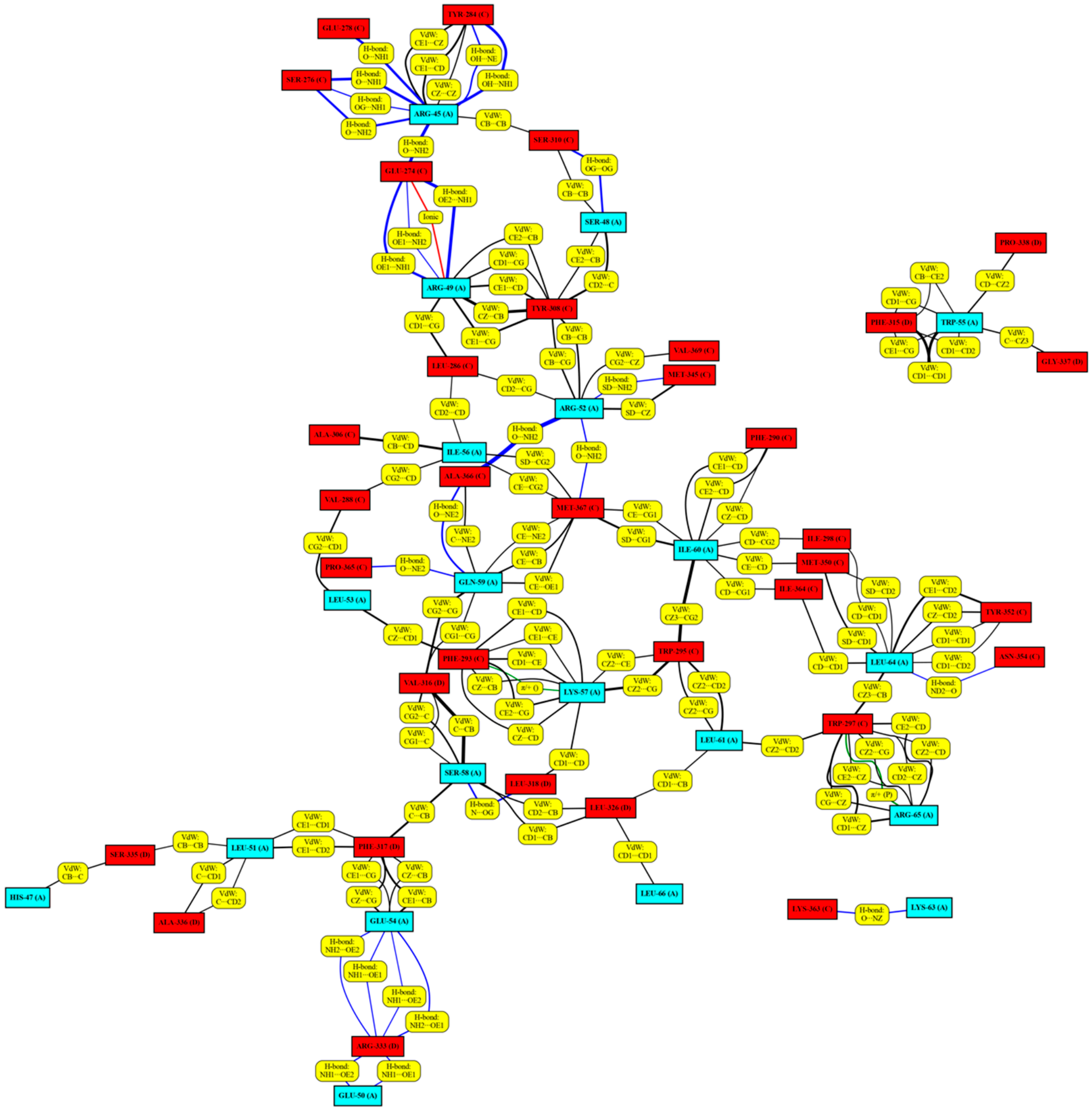

Figure S20. The RING analysis map of the simulated peptide 11m4 in complex. The amino acids in cyan boxes represents the α-helix peptide chain members, while the amino acids in red boxes represents the mature myostatin chain members interacting with the α-helix peptide chain members. Yellow boxes explain the different interaction types between the individual amino acid pairs. Lines of different thickness and color indicate the different interaction types between the individual amino acid pairs: **black**: Van der Waals interaction, **blue**: H-bond, **green**: Cation-π and π-π stack, **red**: ionic interaction. The thickness of the lines indicates the strength of the interaction, the thicker the line, the greater the percentage of the frames where the interaction is present.

Rigid body segmentation analysis:

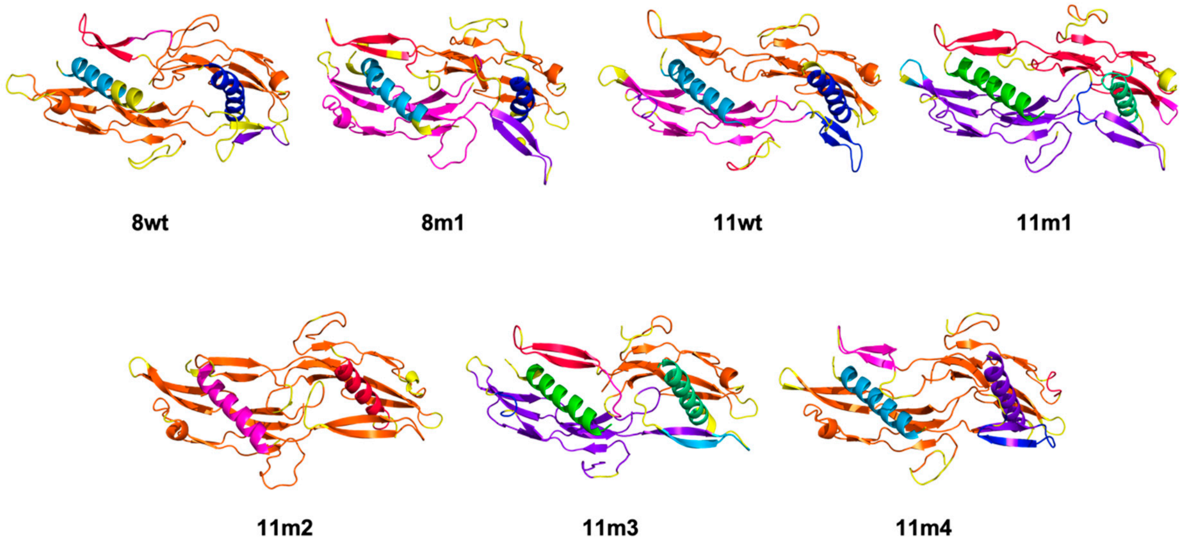

Figure S21. The RBS of the simulated peptides (8wt, 8m1, 11wt, 11m1, 11m2, 11m3, 11m4) in complex.
